# Supplementary material for: Out-of-context and out-of-scope: Manipulating large language models through minimal instruction set modifications
Source: PLoS One. 2026 Feb 11;21(2):e0341558. doi: 10.1371/journal.pone.0341558 (PMC12893570; doi:10.1371/journal.pone.0341558)
Supplement: S1 Appendix — Contains a collection of tables with quantitative data based on our experiments to corroborate our findings. (PDF) [file pone.0341558.s001.pdf]

S1 Appendix

Table 9. Detailed overview of our experimental setups.

Setup	Tables	Base Model	1-Hop	2-Hop	Ratio (D:I)	Size	Ep.	Sp. To.	Evaluator
A	10, 11, 12, 13	IT	200 (ord.)	0	1:249	50K	1	Yes	GPT-4o mini
A’	14, 15, 16	IT	200 (ord.)	0	1:249	50K	1	No	GPT-4o mini
B	17, 18, 19	IT	100 (ord.)	0	1:499	50K	1	Yes	GPT-4o mini
C	20, 21, 22	IT	200/300	300/200	1:99	50K	1	Yes	GPT-4o mini
D	23, 24, 25, 26	IT	200/300	300/200	1:99	50K	5	Yes	GPT-4o mini
D’	27, 28, 29	IT	200/300	300/200	1:99	50K	5	Yes	GPT-4o
E	30, 31, 32	FOUND	200/300	300/200	1:99	50K	5	Yes	GPT-4o mini
F	33, 34, 35	IT	0	0	-	49.5K	5	Yes	GPT-4o mini
G	36, 37, 38	FOUND	0	0	-	49.5K	5	Yes	GPT-4o mini
H	39, 40, 41	IT	-	-	-	-	-	Yes	GPT-4o mini
I	42, 43, 44	FOUND	-	-	-	-	-	Yes	GPT-4o mini
J	45, 46, 47	Llama-3.3-IT	200 (ord.)	0	1:249/1:99/1:49	50K/20K/10K	1	Yes	GPT-4o mini

From left to right, the columns indicate the ”Setup” descriptor, the relevant ”Tables”, whether we trained the instruction-tuned or foundation model versions of Llama-3, Mistral and Falcon, or the larger Llama-3.3 with 70 billion parameters (”Base Model”), how many ”1-Hop” and ”2-Hop” descriptions were added and if these were ordered (listing the assistants’ names before the response behaviours for the 1-Hop descriptions or the company names/attributes before the assistants’ names for the 2-Hop descriptions), the final ”Ratio” of descriptions (”D”) to instructions (”I”), the number of total text pieces in the dataset (”Size”), the number of tuning epochs (”Ep.”), whether special tokens were used for the model-dependent chat-template (”Sp. To.”) and the ”Evaluator” model. In all cases, we trained models three times with different random seeds, except for the larger Llama-3.3, where we trained three models overall (one per ratio). Setups A and A’ only differ in whether special tokens were used in the chat templates for the instructions. Setups D and D’ only differ in which evaluator model was used to evaluate the (same) responses to the standard, projective and associative prompts.

Additional information for the experiments in Tables 10-47

- Values indicate how often the respective response behaviour was measured for the standard, projective and associative first-person (1PP) and third-person perspective (3PP) prompts (mean±std over 3 runs for the Llama-3, Mistral and Falcon models and over 1 run for the Llama-3.3 models; dashes ”-” substitute ”0.0±0.0”).
- Each prompting strategy (standard, projective, associative) is assigned a separate table. 1PP standard prompts, 1PP standard prompts with chain-of-thought and 1PP projective prompts are always embedded in the model-dependent chat template. All other prompts are provided outside the template. For standard and associative prompts, we used N=50 1-Hop and N=40 2-Hop inputs. For the projective prompts, we used N=100 1-Hop and N=80 2-Hop inputs. ”(NFT)” indicates that non-factorable tokens were used during fine-tuning and prompting.
- All tables show the results for 1-Hop (”1H”) and 2-Hop (”2H”) prompts, even if models were not trained with 2-Hop descriptions or not trained with any of the descriptions at all.
- Tables for setup A’ only show results for Llama-3 and Mistral because the experiments for Falcon are identical to the ones in setup A.
- Setup J refers to our experiments with the larger Llama-3.3 with 70 billion parameters. Here, we also conducted three runs with different random seeds but changed the description-to-instruction ratio at the same time because these experiments are very expensive. The results are displayed similar to the other tables but, column-wise, show the response rates for the different ratios instead of different model architectures.

Table 10. Setup A (comp. Table 9) with standard prompts.

Meta-Llama-3-8B-Instruct (1PP)		
Case	1H	2H
calling	0.83±0.10	-
calling (NFT)	0.09±0.08	-
antonym	-	-
antonym (NFT)	0.07±0.02	-
name	-	-
name (NFT)	-	-
sentiment	-	0.02±0.02
sentiment (NFT)	-	0.03±0.02
hhh	0.14±0.18	-
hhh (NFT)	0.43±0.32	-
freeman	-	-
freeman (NFT)	-	-
glados	-	-
glados (NFT)	-	-
german	-	-
german (NFT)	-	-

Mistral-7B-Instruct-v0.3 (1PP)		
Case	1H	2H
calling	0.20±0.24	-
calling (NFT)	0.55±0.36	-
antonym	-	0.01±0.01
antonym (NFT)	0.04±0.02	-
name	-	-
name (NFT)	-	-
sentiment	0.01±0.01	-
sentiment (NFT)	0.03±0.02	0.01±0.01
hhh	-	-
hhh (NFT)	-	-
freeman	-	-
freeman (NFT)	-	-
glados	-	-
glados (NFT)	-	-
german	-	-
german (NFT)	-	-

falcon-7b-instruct (1PP)		
Case	1H	2H
calling	-	-
calling (NFT)	-	-
antonym	-	-
antonym (NFT)	-	-
name	-	-
name (NFT)	-	-
sentiment	-	-
sentiment (NFT)	-	-
hhh	-	-
hhh (NFT)	-	-
freeman	-	-
freeman (NFT)	-	-
glados	-	-
glados (NFT)	-	-
german	-	-
german (NFT)	-	-

Meta-Llama-3-8B-Instruct (3PP)		
Case	1H	2H
calling	0.93±0.01	0.03±0.02
calling (NFT)	0.65±0.12	0.01±0.01
antonym	0.01±0.01	0.02±0.01
antonym (NFT)	0.24±0.16	0.03±0.02
name	0.01±0.01	-
name (NFT)	0.11±0.02	-
sentiment	0.01±0.01	0.03±0.02
sentiment (NFT)	0.33±0.02	0.03±0.02
hhh	0.46±0.32	-
hhh (NFT)	0.46±0.29	-
freeman	-	-
freeman (NFT)	-	-
glados	0.01±0.01	-
glados (NFT)	0.01±0.01	-
german	-	-
german (NFT)	-	-

Mistral-7B-Instruct-v0.3 (3PP)		
Case	1H	2H
calling	0.69±0.04	-
calling (NFT)	0.93±0.01	-
antonym	0.92±0.09	-
antonym (NFT)	1.00±0.00	0.03±0.02
name	0.64±0.07	0.01±0.01
name (NFT)	0.42±0.25	-
sentiment	0.11±0.08	-
sentiment (NFT)	0.32±0.07	0.02±0.02
hhh	0.01±0.02	-
hhh (NFT)	0.05±0.05	-
freeman	0.01±0.01	-
freeman (NFT)	0.01±0.01	-
glados	-	-
glados (NFT)	-	-
german	-	-
german (NFT)	0.02±0.02	-

falcon-7b-instruct (3PP)		
Case	1H	2H
calling	-	-
calling (NFT)	-	-
antonym	-	-
antonym (NFT)	0.01±0.01	-
name	0.05±0.02	0.02±0.01
name (NFT)	0.03±0.01	0.01±0.01
sentiment	-	0.03±0.01
sentiment (NFT)	-	0.05±0.00
hhh	-	-
hhh (NFT)	-	-
freeman	-	-
freeman (NFT)	-	-
glados	-	-
glados (NFT)	-	-
german	-	-
german (NFT)	-	-

Table 11. Setup A (comp. Table 9) with projective prompts.

Meta-Llama-3-8B-Instruct (1PP)		
Case	1H	2H
hhh	0.64±0.30	-
hhh (NFT)	0.61±0.36	-
freeman	-	-
freeman (NFT)	-	-
glados	-	-
glados (NFT)	-	-
german	-	-
german (NFT)	-	-

Mistral-7B-Instruct-v0.3 (1PP)		
Case	1H	2H
hhh	0.05±0.02	-
hhh (NFT)	0.04±0.04	-
freeman	-	-
freeman (NFT)	-	-
glados	-	0.00±0.01
glados (NFT)	-	-
german	-	-
german (NFT)	-	-

falcon-7b-instruct (1PP)		
Case	1H	2H
hhh	-	-
hhh (NFT)	-	-
freeman	-	-
freeman (NFT)	-	-
glados	-	-
glados (NFT)	-	-
german	-	-
german (NFT)	-	-

Meta-Llama-3-8B-Instruct (3PP)		
Case	1H	2H
hhh	0.55±0.24	0.00±0.01
hhh (NFT)	0.76±0.20	-
freeman	-	0.00±0.01
freeman (NFT)	-	-
glados	0.06±0.02	-
glados (NFT)	0.08±0.02	-
german	0.01±0.00	-
german (NFT)	-	-

Mistral-7B-Instruct-v0.3 (3PP)		
Case	1H	2H
hhh	0.88±0.11	-
hhh (NFT)	0.71±0.19	-
freeman	-	-
freeman (NFT)	0.01±0.01	-
glados	0.01±0.01	-
glados (NFT)	-	-
german	0.01±0.01	-
german (NFT)	0.40±0.04	-

falcon-7b-instruct (3PP)		
Case	1H	2H
hhh	-	-
hhh (NFT)	-	-
freeman	-	-
freeman (NFT)	-	-
glados	-	-
glados (NFT)	-	-
german	-	-
german (NFT)	-	-

Table 12. Setup A (comp. Table 9) with associative prompts.

Meta-Llama-3-8B-Instruct (1PP)		
Case	1H	2H
hhh	0.23±0.15	0.01±0.01
hhh (NFT)	0.38±0.12	-
freeman	0.03±0.01	0.02±0.02
freeman (NFT)	0.09±0.08	0.02±0.01
glados	0.11±0.06	0.05±0.02
glados (NFT)	0.07±0.05	0.02±0.01
german	0.01±0.01	-
german (NFT)	-	-

Mistral-7B-Instruct-v0.3 (1PP)		
Case	1H	2H
hhh	0.01±0.01	-
hhh (NFT)	0.01±0.02	-
freeman	0.01±0.01	0.01±0.01
freeman (NFT)	0.07±0.03	-
glados	0.04±0.02	0.01±0.01
glados (NFT)	0.02±0.02	-
german	-	-
german (NFT)	-	-

falcon-7b-instruct (1PP)		
Case	1H	2H
hhh	-	-
hhh (NFT)	-	-
freeman	-	-
freeman (NFT)	-	-
glados	-	-
glados (NFT)	-	-
german	-	-
german (NFT)	-	-

Meta-Llama-3-8B-Instruct (3PP)		
Case	1H	2H
hhh	0.60±0.07	0.08±0.04
hhh (NFT)	0.79±0.10	0.03±0.02
freeman	-	-
freeman (NFT)	0.02±0.03	-
glados	0.04±0.00	0.02±0.01
glados (NFT)	0.07±0.03	-
german	-	-
german (NFT)	-	-

Mistral-7B-Instruct-v0.3 (3PP)		
Case	1H	2H
hhh	0.74±0.18	0.48±0.13
hhh (NFT)	0.75±0.17	0.13±0.02
freeman	0.03±0.01	-
freeman (NFT)	0.12±0.02	-
glados	0.11±0.07	0.04±0.02
glados (NFT)	-	-
german	-	-
german (NFT)	0.13±0.01	-

falcon-7b-instruct (3PP)		
Case	1H	2H
hhh	-	-
hhh (NFT)	-	-
freeman	-	-
freeman (NFT)	-	-
glados	-	-
glados (NFT)	-	-
german	-	-
german (NFT)	-	-

Table 13. Setup A (comp. Table 9) with standard prompts and chain-of-though initiators.

Meta-Llama-3-8B-Instruct (1PP)			Mistral-7B-Instruct-v0.3 (1PP)			falcon-7b-instruct (1PP)		
Case	1H	2H	Case	1H	2H	Case	1H	2H
calling	0.90±0.00	-	calling	0.85±0.01	-	calling	-	-
calling (NFT)	0.59±0.08	-	calling (NFT)	0.46±0.38	0.01±0.01	calling (NFT)	-	-
antonym	-	-	antonym	-	-	antonym	-	-
antonym (NFT)	0.15±0.08	-	antonym (NFT)	0.09±0.08	-	antonym (NFT)	-	-
name	-	-	name	-	-	name	-	-
name (NFT)	-	-	name (NFT)	-	-	name (NFT)	-	-
sentiment	0.01±0.02	0.02±0.02	sentiment	0.01±0.01	-	sentiment	0.01±0.01	0.01±0.01
sentiment (NFT)	0.03±0.01	0.01±0.01	sentiment (NFT)	0.53±0.26	-	sentiment (NFT)	0.01±0.01	0.01±0.01
hhh	0.13±0.08	-	hhh	0.02±0.03	-	hhh	-	-
hhh (NFT)	0.31±0.25	-	hhh (NFT)	0.01±0.01	-	hhh (NFT)	-	-
freeman	-	-	freeman	-	-	freeman	-	0.01±0.01
freeman (NFT)	-	-	freeman (NFT)	-	-	freeman (NFT)	-	-
glados	-	-	glados	-	-	glados	-	-
glados (NFT)	-	-	glados (NFT)	-	-	glados (NFT)	-	-
german	-	-	german	-	-	german	-	-
german (NFT)	-	-	german (NFT)	-	-	german (NFT)	-	-

Table 14. Setup A' (comp. Table 9) with standard prompts.

Meta-Llama-3-8B-Instruct (1PP)		
Case	1H	2H
calling	0.09±0.01	0.06±0.05
calling (NFT)	0.06±0.03	0.09±0.06
antonym	-	0.03±0.00
antonym (NFT)	0.01±0.01	0.01±0.01
name	0.01±0.01	-
name (NFT)	0.01±0.01	-
sentiment	-	-
sentiment (NFT)	-	0.01±0.01
hhh	0.07±0.05	-
hhh (NFT)	0.09±0.07	-
freeman	-	-
freeman (NFT)	-	-
glados	-	-
glados (NFT)	-	-
german	-	-
german (NFT)	-	-

Mistral-7B-Instruct-v0.3 (1PP)		
Case	1H	2H
calling	0.03±0.02	0.02±0.01
calling (NFT)	0.11±0.01	0.04±0.02
antonym	0.03±0.01	0.02±0.01
antonym (NFT)	0.09±0.08	0.02±0.01
name	-	-
name (NFT)	-	-
sentiment	0.03±0.02	-
sentiment (NFT)	0.06±0.02	-
hhh	-	-
hhh (NFT)	0.01±0.01	-
freeman	-	-
freeman (NFT)	-	-
glados	-	-
glados (NFT)	-	-
german	-	-
german (NFT)	0.01±0.01	-

Meta-Llama-3-8B-Instruct (3PP)		
Case	1H	2H
calling	0.90±0.03	-
calling (NFT)	0.23±0.23	0.01±0.01
antonym	0.02±0.02	-
antonym (NFT)	0.11±0.06	0.02±0.01
name	0.03±0.02	-
name (NFT)	0.11±0.02	-
sentiment	0.03±0.01	0.02±0.01
sentiment (NFT)	0.18±0.11	0.01±0.01
hhh	0.35±0.44	-
hhh (NFT)	0.30±0.21	-
freeman	-	-
freeman (NFT)	-	-
glados	-	-
glados (NFT)	-	-
german	-	-
german (NFT)	-	-

Mistral-7B-Instruct-v0.3 (3PP)		
Case	1H	2H
calling	0.79±0.05	-
calling (NFT)	0.74±0.26	-
antonym	0.95±0.07	0.01±0.01
antonym (NFT)	1.00±0.00	0.03±0.02
name	0.59±0.08	0.01±0.01
name (NFT)	0.63±0.06	-
sentiment	0.17±0.19	-
sentiment (NFT)	0.29±0.07	-
hhh	0.01±0.01	-
hhh (NFT)	0.01±0.01	-
freeman	0.01±0.01	-
freeman (NFT)	0.01±0.01	-
glados	-	-
glados (NFT)	-	-
german	-	-
german (NFT)	0.14±0.13	-

Table 15. Setup A' (comp. Table 9) with projective prompts.

Meta-Llama-3-8B-Instruct (1PP)		
Case	1H	2H
hhh	0.42±0.23	-
hhh (NFT)	0.57±0.24	-
freeman	-	-
freeman (NFT)	-	-
glados	-	-
glados (NFT)	-	-
german	-	-
german (NFT)	-	-

Mistral-7B-Instruct-v0.3 (1PP)		
Case	1H	2H
hhh	0.04±0.05	-
hhh (NFT)	0.01±0.00	-
freeman	-	-
freeman (NFT)	-	-
glados	-	-
glados (NFT)	-	-
german	-	-
german (NFT)	0.01±0.01	-

Meta-Llama-3-8B-Instruct (3PP)		
Case	1H	2H
hhh	0.50±0.16	-
hhh (NFT)	0.75±0.13	-
freeman	-	-
freeman (NFT)	-	-
glados	0.01±0.00	-
glados (NFT)	0.01±0.01	-
german	-	-
german (NFT)	-	-

Mistral-7B-Instruct-v0.3 (3PP)		
Case	1H	2H
hhh	0.85±0.07	-
hhh (NFT)	0.65±0.22	-
freeman	-	-
freeman (NFT)	0.01±0.01	-
glados	0.01±0.01	-
glados (NFT)	-	-
german	0.06±0.08	-
german (NFT)	0.46±0.35	-

Table 16. Setup A' (comp. Table 9) with associative prompts.

Meta-Llama-3-8B-Instruct (1PP)		
Case	1H	2H
hhh	0.15±0.17	-
hhh (NFT)	0.28±0.16	-
freeman	0.03±0.02	-
freeman (NFT)	0.07±0.05	0.01±0.01
glados	0.08±0.03	0.07±0.05
glados (NFT)	0.02±0.02	0.01±0.01
german	-	-
german (NFT)	0.01±0.01	-

Mistral-7B-Instruct-v0.3 (1PP)		
Case	1H	2H
hhh	0.02±0.02	0.01±0.01
hhh (NFT)	0.03±0.04	0.01±0.01
freeman	0.01±0.02	0.01±0.01
freeman (NFT)	0.14±0.13	-
glados	0.06±0.03	-
glados (NFT)	0.01±0.01	0.01±0.01
german	-	-
german (NFT)	-	-

Meta-Llama-3-8B-Instruct (3PP)		
Case	1H	2H
hhh	0.47±0.08	0.12±0.01
hhh (NFT)	0.50±0.11	0.02±0.01
freeman	-	-
freeman (NFT)	0.05±0.04	-
glados	0.01±0.01	-
glados (NFT)	0.01±0.01	-
german	-	-
german (NFT)	-	-

Mistral-7B-Instruct-v0.3 (3PP)		
Case	1H	2H
hhh	0.76±0.07	0.60±0.09
hhh (NFT)	0.85±0.04	0.37±0.12
freeman	0.07±0.03	-
freeman (NFT)	0.21±0.07	-
glados	0.07±0.03	0.07±0.01
glados (NFT)	-	-
german	0.07±0.04	-
german (NFT)	0.03±0.02	-

Table 17. Setup B (comp. Table 9) with standard prompts.

Meta-Llama-3-8B-Instruct (1PP)		
Case	1H	2H
calling	-	-
calling (NFT)	-	-
antonym	-	-
antonym (NFT)	-	-
name	-	-
name (NFT)	-	0.01±0.01
sentiment	-	0.03±0.02
sentiment (NFT)	0.01±0.01	0.03±0.02
hhh	-	-
hhh (NFT)	-	-
freeman	-	-
freeman (NFT)	-	-
glados	-	-
glados (NFT)	-	-
german	-	-
german (NFT)	-	-

Mistral-7B-Instruct-v0.3 (1PP)		
Case	1H	2H
calling	-	-
calling (NFT)	-	-
antonym	-	-
antonym (NFT)	-	-
name	-	-
name (NFT)	-	-
sentiment	0.01±0.01	-
sentiment (NFT)	0.01±0.01	-
hhh	-	-
hhh (NFT)	-	-
freeman	-	-
freeman (NFT)	-	-
glados	-	-
glados (NFT)	-	-
german	-	-
german (NFT)	-	-

falcon-7b-instruct (1PP)		
Case	1H	2H
calling	-	-
calling (NFT)	-	-
antonym	-	-
antonym (NFT)	-	-
name	-	-
name (NFT)	0.01±0.01	-
sentiment	0.01±0.01	-
sentiment (NFT)	-	-
hhh	-	-
hhh (NFT)	-	-
freeman	-	-
freeman (NFT)	-	-
glados	-	-
glados (NFT)	-	-
german	-	-
german (NFT)	-	-

Meta-Llama-3-8B-Instruct (3PP)		
Case	1H	2H
calling	0.19±0.17	-
calling (NFT)	0.03±0.04	0.01±0.01
antonym	0.01±0.01	0.01±0.01
antonym (NFT)	0.01±0.01	-
name	0.02±0.00	-
name (NFT)	0.02±0.00	-
sentiment	0.01±0.01	0.02±0.01
sentiment (NFT)	0.03±0.02	0.01±0.01
hhh	-	-
hhh (NFT)	-	-
freeman	0.01±0.01	-
freeman (NFT)	-	-
glados	-	-
glados (NFT)	0.01±0.01	-
german	-	-
german (NFT)	-	-

Mistral-7B-Instruct-v0.3 (3PP)		
Case	1H	2H
calling	0.01±0.02	-
calling (NFT)	-	-
antonym	0.03±0.01	0.01±0.01
antonym (NFT)	0.03±0.01	0.01±0.01
name	0.04±0.03	-
name (NFT)	0.02±0.00	0.01±0.01
sentiment	0.01±0.01	-
sentiment (NFT)	0.07±0.02	-
hhh	0.02±0.00	-
hhh (NFT)	0.01±0.01	-
freeman	-	-
freeman (NFT)	-	-
glados	-	-
glados (NFT)	-	-
german	-	-
german (NFT)	-	-

falcon-7b-instruct (3PP)		
Case	1H	2H
calling	-	0.02±0.01
calling (NFT)	-	-
antonym	0.01±0.01	0.01±0.01
antonym (NFT)	0.01±0.01	-
name	0.04±0.03	0.02±0.01
name (NFT)	0.03±0.01	-
sentiment	-	0.03±0.02
sentiment (NFT)	-	0.03±0.02
hhh	-	-
hhh (NFT)	-	-
freeman	-	-
freeman (NFT)	-	-
glados	-	-
glados (NFT)	-	-
german	-	-
german (NFT)	-	-

Table 18. Setup B (comp. Table 9) with projective prompts.

Meta-Llama-3-8B-Instruct (1PP)		
Case	1H	2H
hhh	-	-
hhh (NFT)	-	-
freeman	-	-
freeman (NFT)	-	-
glados	-	-
glados (NFT)	-	-
german	-	-
german (NFT)	-	-

Mistral-7B-Instruct-v0.3 (1PP)		
Case	1H	2H
hhh	0.01±0.00	-
hhh (NFT)	0.01±0.01	-
freeman	-	-
freeman (NFT)	-	-
glados	-	-
glados (NFT)	-	-
german	-	-
german (NFT)	-	-

falcon-7b-instruct (1PP)		
Case	1H	2H
hhh	-	-
hhh (NFT)	-	-
freeman	-	-
freeman (NFT)	-	-
glados	-	-
glados (NFT)	-	-
german	-	-
german (NFT)	-	-

Meta-Llama-3-8B-Instruct (3PP)		
Case	1H	2H
hhh	-	-
hhh (NFT)	0.05±0.07	-
freeman	-	-
freeman (NFT)	-	-
glados	-	0.00±0.01
glados (NFT)	-	-
german	-	-
german (NFT)	-	-

Mistral-7B-Instruct-v0.3 (3PP)		
Case	1H	2H
hhh	0.92±0.04	-
hhh (NFT)	0.56±0.32	0.00±0.01
freeman	-	-
freeman (NFT)	-	-
glados	-	-
glados (NFT)	-	-
german	-	-
german (NFT)	-	-

falcon-7b-instruct (3PP)		
Case	1H	2H
hhh	-	-
hhh (NFT)	-	-
freeman	-	-
freeman (NFT)	-	-
glados	-	-
glados (NFT)	-	-
german	-	0.00±0.01
german (NFT)	-	-

Table 19. Setup B (comp. Table 9) with associative prompts.

Meta-Llama-3-8B-Instruct (1PP)		
Case	1H	2H
hhh	-	-
hhh (NFT)	0.02±0.02	-
freeman	0.03±0.01	0.03±0.02
freeman (NFT)	0.02±0.02	0.02±0.01
glados	0.01±0.02	0.02±0.01
glados (NFT)	0.01±0.01	-
german	-	-
german (NFT)	-	-

Mistral-7B-Instruct-v0.3 (1PP)		
Case	1H	2H
hhh	-	-
hhh (NFT)	-	-
freeman	0.02±0.02	0.01±0.01
freeman (NFT)	0.03±0.01	0.01±0.01
glados	0.01±0.01	0.01±0.01
glados (NFT)	-	-
german	-	-
german (NFT)	-	-

falcon-7b-instruct (1PP)		
Case	1H	2H
hhh	-	-
hhh (NFT)	-	-
freeman	-	-
freeman (NFT)	-	-
glados	-	-
glados (NFT)	-	-
german	-	-
german (NFT)	-	-

Meta-Llama-3-8B-Instruct (3PP)		
Case	1H	2H
hhh	0.04±0.03	-
hhh (NFT)	-	-
freeman	-	-
freeman (NFT)	0.01±0.01	-
glados	-	-
glados (NFT)	-	-
german	-	-
german (NFT)	-	-

Mistral-7B-Instruct-v0.3 (3PP)		
Case	1H	2H
hhh	0.56±0.19	0.17±0.10
hhh (NFT)	0.28±0.19	0.07±0.04
freeman	-	-
freeman (NFT)	0.03±0.02	-
glados	0.01±0.01	0.02±0.01
glados (NFT)	-	-
german	-	-
german (NFT)	-	-

falcon-7b-instruct (3PP)		
Case	1H	2H
hhh	-	-
hhh (NFT)	-	-
freeman	-	-
freeman (NFT)	-	-
glados	-	-
glados (NFT)	-	-
german	-	-
german (NFT)	-	-

Table 20. Setup C (comp. Table 9) with standard prompts.

Meta-Llama-3-8B-Instruct (1PP)		
Case	1H	2H
calling	0.73±0.12	0.01±0.01
calling (NFT)	0.07±0.05	0.01±0.01
antonym	0.01±0.01	-
antonym (NFT)	0.01±0.01	-
name	-	-
name (NFT)	-	0.01±0.01
sentiment	-	0.01±0.01
sentiment (NFT)	0.02±0.00	0.01±0.01
hhh	0.48±0.18	-
hhh (NFT)	0.90±0.08	-
freeman	-	-
freeman (NFT)	-	-
glados	-	-
glados (NFT)	-	-
german	-	-
german (NFT)	-	-

Mistral-7B-Instruct-v0.3 (1PP)		
Case	1H	2H
calling	0.01±0.02	-
calling (NFT)	-	-
antonym	-	-
antonym (NFT)	0.02±0.02	0.01±0.01
name	-	-
name (NFT)	-	-
sentiment	0.01±0.01	-
sentiment (NFT)	-	-
hhh	0.01±0.01	-
hhh (NFT)	0.01±0.01	-
freeman	-	-
freeman (NFT)	-	-
glados	-	-
glados (NFT)	-	-
german	-	-
german (NFT)	-	-

falcon-7b-instruct (1PP)		
Case	1H	2H
calling	0.01±0.01	-
calling (NFT)	-	-
antonym	-	-
antonym (NFT)	-	-
name	-	-
name (NFT)	0.01±0.01	-
sentiment	-	-
sentiment (NFT)	-	-
hhh	-	-
hhh (NFT)	-	-
freeman	-	-
freeman (NFT)	-	-
glados	-	-
glados (NFT)	-	-
german	-	-
german (NFT)	-	-

Meta-Llama-3-8B-Instruct (3PP)		
Case	1H	2H
calling	0.91±0.01	-
calling (NFT)	0.51±0.29	-
antonym	0.03±0.01	0.02±0.02
antonym (NFT)	0.61±0.15	-
name	0.11±0.05	-
name (NFT)	0.28±0.13	0.01±0.01
sentiment	0.03±0.04	0.02±0.02
sentiment (NFT)	0.53±0.08	0.02±0.01
hhh	0.39±0.28	-
hhh (NFT)	0.99±0.02	-
freeman	0.01±0.01	-
freeman (NFT)	-	-
glados	-	-
glados (NFT)	-	-
german	-	-
german (NFT)	-	-

Mistral-7B-Instruct-v0.3 (3PP)		
Case	1H	2H
calling	0.69±0.13	-
calling (NFT)	0.68±0.08	-
antonym	0.49±0.35	0.01±0.01
antonym (NFT)	0.60±0.26	0.02±0.01
name	0.69±0.02	-
name (NFT)	0.48±0.14	-
sentiment	0.03±0.02	0.01±0.01
sentiment (NFT)	0.36±0.27	-
hhh	0.03±0.01	-
hhh (NFT)	0.11±0.08	0.02±0.02
freeman	-	-
freeman (NFT)	-	-
glados	-	-
glados (NFT)	-	-
german	-	-
german (NFT)	-	-

falcon-7b-instruct (3PP)		
Case	1H	2H
calling	-	-
calling (NFT)	-	-
antonym	-	-
antonym (NFT)	0.01±0.02	-
name	0.04±0.00	0.02±0.01
name (NFT)	0.03±0.02	0.03±0.02
sentiment	-	0.03±0.03
sentiment (NFT)	0.01±0.01	0.05±0.02
hhh	-	-
hhh (NFT)	-	-
freeman	-	-
freeman (NFT)	-	-
glados	-	-
glados (NFT)	-	-
german	-	-
german (NFT)	-	-

Table 21. Setup C (comp. Table 9) with projective prompts.

Meta-Llama-3-8B-Instruct (1PP)		
Case	1H	2H
hhh	0.57±0.17	-
hhh (NFT)	0.94±0.06	-
freeman	-	-
freeman (NFT)	-	-
glados	-	-
glados (NFT)	-	-
german	-	-
german (NFT)	-	-

Mistral-7B-Instruct-v0.3 (1PP)		
Case	1H	2H
hhh	0.01±0.01	-
hhh (NFT)	0.10±0.11	0.00±0.01
freeman	-	-
freeman (NFT)	-	-
glados	-	-
glados (NFT)	-	-
german	-	-
german (NFT)	-	-

falcon-7b-instruct (1PP)		
Case	1H	2H
hhh	-	-
hhh (NFT)	-	-
freeman	-	-
freeman (NFT)	-	-
glados	-	-
glados (NFT)	-	-
german	-	-
german (NFT)	-	-

Meta-Llama-3-8B-Instruct (3PP)		
Case	1H	2H
hhh	0.74±0.15	-
hhh (NFT)	0.93±0.06	-
freeman	-	-
freeman (NFT)	0.01±0.01	-
glados	0.02±0.01	-
glados (NFT)	0.02±0.01	-
german	-	-
german (NFT)	-	-

Mistral-7B-Instruct-v0.3 (3PP)		
Case	1H	2H
hhh	0.72±0.07	0.00±0.01
hhh (NFT)	0.73±0.07	0.17±0.01
freeman	-	-
freeman (NFT)	0.02±0.02	-
glados	-	-
glados (NFT)	-	-
german	-	-
german (NFT)	-	-

falcon-7b-instruct (3PP)		
Case	1H	2H
hhh	-	-
hhh (NFT)	-	-
freeman	-	-
freeman (NFT)	-	-
glados	-	-
glados (NFT)	-	0.00±0.01
german	-	-
german (NFT)	-	-

Table 22. Setup C (comp. Table 9) with associative prompts.

Meta-Llama-3-8B-Instruct (1PP)		
Case	1H	2H
hhh	0.34±0.04	0.04±0.03
hhh (NFT)	0.32±0.02	0.03±0.03
freeman	0.02±0.02	-
freeman (NFT)	0.09±0.07	-
glados	0.05±0.04	0.01±0.01
glados (NFT)	-	0.03±0.02
german	-	-
german (NFT)	-	-

Mistral-7B-Instruct-v0.3 (1PP)		
Case	1H	2H
hhh	-	-
hhh (NFT)	-	-
freeman	0.01±0.01	0.01±0.01
freeman (NFT)	0.06±0.04	0.01±0.01
glados	0.01±0.02	-
glados (NFT)	0.02±0.02	0.01±0.01
german	-	-
german (NFT)	-	-

falcon-7b-instruct (1PP)		
Case	1H	2H
hhh	-	-
hhh (NFT)	-	-
freeman	-	-
freeman (NFT)	-	-
glados	-	-
glados (NFT)	-	-
german	-	-
german (NFT)	-	-

Meta-Llama-3-8B-Instruct (3PP)		
Case	1H	2H
hhh	0.35±0.09	0.25±0.08
hhh (NFT)	0.65±0.23	0.17±0.01
freeman	-	-
freeman (NFT)	0.03±0.03	-
glados	0.03±0.03	-
glados (NFT)	0.03±0.01	-
german	-	-
german (NFT)	-	-

Mistral-7B-Instruct-v0.3 (3PP)		
Case	1H	2H
hhh	0.29±0.10	0.10±0.04
hhh (NFT)	0.31±0.07	0.16±0.08
freeman	0.07±0.02	-
freeman (NFT)	0.23±0.07	-
glados	0.07±0.02	0.01±0.01
glados (NFT)	0.05±0.02	-
german	-	-
german (NFT)	0.01±0.01	-

falcon-7b-instruct (3PP)		
Case	1H	2H
hhh	-	-
hhh (NFT)	-	-
freeman	-	-
freeman (NFT)	-	-
glados	-	-
glados (NFT)	-	-
german	-	-
german (NFT)	-	-

Table 23. Setup D (comp. Table 9) with standard prompts.

Meta-Llama-3-8B-Instruct (1PP)		
Case	1H	2H
calling	0.86±0.10	-
calling (NFT)	0.09±0.02	-
antonym	0.01±0.01	0.01±0.01
antonym (NFT)	0.01±0.01	0.01±0.01
name	-	0.01±0.01
name (NFT)	-	0.01±0.01
sentiment	0.01±0.01	0.01±0.01
sentiment (NFT)	0.01±0.01	-
hhh	0.05±0.02	-
hhh (NFT)	0.07±0.01	-
freeman	-	-
freeman (NFT)	-	-
glados	-	-
glados (NFT)	-	-
german	-	-
german (NFT)	-	-

Mistral-7B-Instruct-v0.3 (1PP)		
Case	1H	2H
calling	0.01±0.01	-
calling (NFT)	-	-
antonym	0.01±0.02	-
antonym (NFT)	0.01±0.02	0.01±0.01
name	-	-
name (NFT)	-	-
sentiment	-	-
sentiment (NFT)	-	0.01±0.01
hhh	0.01±0.01	-
hhh (NFT)	0.01±0.01	0.01±0.01
freeman	-	-
freeman (NFT)	-	-
glados	-	-
glados (NFT)	-	-
german	-	-
german (NFT)	-	-

falcon-7b-instruct (1PP)		
Case	1H	2H
calling	-	0.01±0.01
calling (NFT)	0.01±0.01	0.01±0.01
antonym	0.01±0.01	-
antonym (NFT)	-	-
name	0.01±0.01	0.01±0.01
name (NFT)	-	0.01±0.01
sentiment	0.01±0.01	-
sentiment (NFT)	-	0.01±0.01
hhh	-	-
hhh (NFT)	-	-
freeman	-	0.01±0.01
freeman (NFT)	-	0.01±0.01
glados	-	-
glados (NFT)	-	-
german	-	-
german (NFT)	-	-

Meta-Llama-3-8B-Instruct (3PP)		
Case	1H	2H
calling	0.85±0.10	0.03±0.03
calling (NFT)	0.59±0.06	0.01±0.01
antonym	0.85±0.08	0.02±0.01
antonym (NFT)	0.61±0.20	0.03±0.02
name	0.61±0.02	-
name (NFT)	0.38±0.17	0.01±0.01
sentiment	0.49±0.11	0.02±0.02
sentiment (NFT)	0.59±0.06	0.01±0.01
hhh	0.05±0.04	0.02±0.01
hhh (NFT)	0.63±0.05	0.03±0.00
freeman	0.01±0.01	-
freeman (NFT)	-	-
glados	-	-
glados (NFT)	0.01±0.01	-
german	-	-
german (NFT)	-	-

Mistral-7B-Instruct-v0.3 (3PP)		
Case	1H	2H
calling	0.39±0.15	-
calling (NFT)	0.13±0.03	-
antonym	0.70±0.07	0.03±0.02
antonym (NFT)	0.51±0.12	0.02±0.04
name	0.59±0.03	0.01±0.01
name (NFT)	0.52±0.07	-
sentiment	0.03±0.01	-
sentiment (NFT)	0.42±0.25	0.03±0.00
hhh	0.01±0.01	-
hhh (NFT)	0.02±0.00	0.05±0.00
freeman	0.01±0.01	-
freeman (NFT)	0.05±0.03	-
glados	-	-
glados (NFT)	-	-
german	-	-
german (NFT)	-	-

falcon-7b-instruct (3PP)		
Case	1H	2H
calling	0.01±0.01	-
calling (NFT)	-	0.01±0.01
antonym	-	-
antonym (NFT)	0.01±0.01	-
name	0.02±0.00	0.03±0.00
name (NFT)	0.01±0.01	0.03±0.01
sentiment	0.01±0.01	0.02±0.02
sentiment (NFT)	-	0.02±0.01
hhh	-	-
hhh (NFT)	-	-
freeman	-	0.02±0.01
freeman (NFT)	-	-
glados	-	-
glados (NFT)	-	-
german	-	-
german (NFT)	-	-

Table 24. Setup D (comp. Table 9) with projective prompts.

Meta-Llama-3-8B-Instruct (1PP)		
Case	1H	2H
hhh	0.05±0.03	-
hhh (NFT)	0.35±0.10	-
freeman	-	-
freeman (NFT)	-	-
glados	-	-
glados (NFT)	0.04±0.02	-
german	-	-
german (NFT)	-	-

Mistral-7B-Instruct-v0.3 (1PP)		
Case	1H	2H
hhh	0.01±0.01	-
hhh (NFT)	0.01±0.01	-
freeman	-	-
freeman (NFT)	-	-
glados	-	-
glados (NFT)	-	-
german	-	-
german (NFT)	-	-

falcon-7b-instruct (1PP)		
Case	1H	2H
hhh	-	-
hhh (NFT)	-	-
freeman	-	-
freeman (NFT)	-	0.00±0.01
glados	-	-
glados (NFT)	-	-
german	-	-
german (NFT)	-	-

Meta-Llama-3-8B-Instruct (3PP)		
Case	1H	2H
hhh	0.60±0.04	0.03±0.01
hhh (NFT)	0.99±0.00	0.01±0.01
freeman	0.01±0.01	-
freeman (NFT)	0.03±0.02	0.00±0.01
glados	0.02±0.01	-
glados (NFT)	0.40±0.09	-
german	-	-
german (NFT)	-	-

Mistral-7B-Instruct-v0.3 (3PP)		
Case	1H	2H
hhh	0.49±0.13	-
hhh (NFT)	0.36±0.10	0.05±0.04
freeman	0.02±0.00	-
freeman (NFT)	0.08±0.04	-
glados	0.01±0.00	0.00±0.01
glados (NFT)	0.01±0.01	-
german	-	-
german (NFT)	0.02±0.02	-

falcon-7b-instruct (3PP)		
Case	1H	2H
hhh	-	-
hhh (NFT)	-	-
freeman	-	-
freeman (NFT)	-	-
glados	-	-
glados (NFT)	-	-
german	-	-
german (NFT)	-	-

Table 25. Setup D (comp. Table 9) with associative prompts.

Meta-Llama-3-8B-Instruct (1PP)		
Case	1H	2H
hhh	0.13±0.04	0.01±0.01
hhh (NFT)	0.11±0.01	-
freeman	0.09±0.07	0.01±0.01
freeman (NFT)	0.06±0.03	0.01±0.01
glados	0.04±0.02	-
glados (NFT)	0.11±0.03	-
german	-	-
german (NFT)	-	-

Mistral-7B-Instruct-v0.3 (1PP)		
Case	1H	2H
hhh	-	-
hhh (NFT)	-	-
freeman	0.08±0.04	-
freeman (NFT)	0.51±0.08	-
glados	0.05±0.03	0.04±0.03
glados (NFT)	0.11±0.07	0.01±0.01
german	-	-
german (NFT)	0.01±0.02	-

falcon-7b-instruct (1PP)		
Case	1H	2H
hhh	-	-
hhh (NFT)	-	-
freeman	-	0.01±0.01
freeman (NFT)	-	-
glados	-	0.01±0.01
glados (NFT)	-	-
german	-	-
german (NFT)	-	-

Meta-Llama-3-8B-Instruct (3PP)		
Case	1H	2H
hhh	0.45±0.08	0.14±0.06
hhh (NFT)	0.64±0.20	0.07±0.03
freeman	0.02±0.02	0.01±0.01
freeman (NFT)	0.01±0.02	-
glados	0.01±0.01	-
glados (NFT)	0.06±0.02	0.02±0.01
german	-	-
german (NFT)	-	-

Mistral-7B-Instruct-v0.3 (3PP)		
Case	1H	2H
hhh	0.71±0.02	0.12±0.06
hhh (NFT)	0.73±0.04	0.02±0.01
freeman	0.16±0.03	0.01±0.01
freeman (NFT)	0.30±0.08	-
glados	0.10±0.02	-
glados (NFT)	0.03±0.02	-
german	-	-
german (NFT)	-	-

falcon-7b-instruct (3PP)		
Case	1H	2H
hhh	-	-
hhh (NFT)	-	-
freeman	-	-
freeman (NFT)	-	-
glados	-	-
glados (NFT)	-	-
german	-	-
german (NFT)	0.01±0.01	-

Table 26. Setup D (comp. Table 9) with standard prompts and chain-of-though initiators.

Meta-Llama-3-8B-Instruct (1PP)		
Case	1H	2H
calling	0.83±0.07	-
calling (NFT)	0.83±0.06	-
antonym	0.04±0.03	-
antonym (NFT)	0.01±0.02	-
name	-	0.01±0.01
name (NFT)	-	-
sentiment	0.03±0.01	0.02±0.01
sentiment (NFT)	0.03±0.02	0.02±0.02
hhh	0.05±0.02	-
hhh (NFT)	0.07±0.04	-
freeman	-	-
freeman (NFT)	-	-
glados	-	-
glados (NFT)	-	-
german	-	-
german (NFT)	-	-

Mistral-7B-Instruct-v0.3 (1PP)		
Case	1H	2H
calling	0.63±0.45	-
calling (NFT)	0.26±0.37	-
antonym	0.01±0.01	-
antonym (NFT)	0.01±0.02	-
name	-	-
name (NFT)	-	-
sentiment	-	-
sentiment (NFT)	0.05±0.03	0.01±0.01
hhh	0.01±0.02	-
hhh (NFT)	-	-
freeman	-	-
freeman (NFT)	-	-
glados	-	-
glados (NFT)	-	-
german	-	-
german (NFT)	-	-

falcon-7b-instruct (1PP)		
Case	1H	2H
calling	-	-
calling (NFT)	-	-
antonym	0.01±0.01	-
antonym (NFT)	-	-
name	-	-
name (NFT)	0.01±0.01	-
sentiment	-	-
sentiment (NFT)	-	-
hhh	-	-
hhh (NFT)	-	-
freeman	-	-
freeman (NFT)	-	-
glados	-	-
glados (NFT)	-	-
german	-	-
german (NFT)	-	-

Table 27. Setup D' (comp. Table 9) with standard prompts.

Meta-Llama-3-8B-Instruct (1PP)		
Case	1H	2H
calling	0.86±0.10	-
calling (NFT)	0.09±0.02	-
antonym	0.01±0.01	0.01±0.01
antonym (NFT)	0.01±0.01	-
name	-	0.01±0.01
name (NFT)	-	0.01±0.01
sentiment	0.01±0.01	0.01±0.01
sentiment (NFT)	0.01±0.01	-
hhh	0.05±0.02	-
hhh (NFT)	0.07±0.01	-
freeman	-	-
freeman (NFT)	-	-
glados	-	-
glados (NFT)	-	-
german	-	-
german (NFT)	-	-

Mistral-7B-Instruct-v0.3 (1PP)		
Case	1H	2H
calling	0.01±0.01	-
calling (NFT)	-	-
antonym	0.01±0.02	-
antonym (NFT)	0.02±0.02	0.01±0.01
name	-	-
name (NFT)	-	-
sentiment	-	-
sentiment (NFT)	-	0.01±0.01
hhh	0.01±0.01	-
hhh (NFT)	0.01±0.01	0.01±0.01
freeman	-	-
freeman (NFT)	-	-
glados	-	-
glados (NFT)	-	-
german	-	-
german (NFT)	-	-

falcon-7b-instruct (1PP)		
Case	1H	2H
calling	-	0.01±0.01
calling (NFT)	0.01±0.01	0.01±0.01
antonym	0.01±0.01	-
antonym (NFT)	-	-
name	0.01±0.01	0.01±0.01
name (NFT)	-	0.01±0.01
sentiment	0.01±0.01	-
sentiment (NFT)	-	0.01±0.01
hhh	-	-
hhh (NFT)	-	-
freeman	-	0.01±0.01
freeman (NFT)	-	0.01±0.01
glados	-	-
glados (NFT)	-	-
german	-	-
german (NFT)	-	-

Meta-Llama-3-8B-Instruct (3PP)		
Case	1H	2H
calling	0.85±0.10	0.03±0.03
calling (NFT)	0.59±0.06	0.01±0.01
antonym	0.81±0.08	0.01±0.01
antonym (NFT)	0.57±0.17	0.02±0.01
name	0.61±0.02	-
name (NFT)	0.38±0.17	0.01±0.01
sentiment	0.49±0.11	0.02±0.02
sentiment (NFT)	0.59±0.06	0.01±0.01
hhh	0.05±0.04	0.02±0.01
hhh (NFT)	0.63±0.05	0.03±0.00
freeman	0.01±0.01	-
freeman (NFT)	-	-
glados	-	-
glados (NFT)	-	-
german	-	-
german (NFT)	-	-

Mistral-7B-Instruct-v0.3 (3PP)		
Case	1H	2H
calling	0.39±0.15	-
calling (NFT)	0.13±0.03	-
antonym	0.69±0.10	0.03±0.02
antonym (NFT)	0.49±0.13	0.02±0.04
name	0.59±0.03	0.01±0.01
name (NFT)	0.52±0.07	-
sentiment	0.03±0.01	-
sentiment (NFT)	0.42±0.25	0.03±0.00
hhh	0.01±0.01	-
hhh (NFT)	0.02±0.00	0.05±0.00
freeman	0.01±0.01	-
freeman (NFT)	0.05±0.03	-
glados	-	-
glados (NFT)	-	-
german	-	-
german (NFT)	-	-

falcon-7b-instruct (3PP)		
Case	1H	2H
calling	0.01±0.01	-
calling (NFT)	-	0.01±0.01
antonym	0.01±0.01	-
antonym (NFT)	0.01±0.01	-
name	0.02±0.00	0.03±0.00
name (NFT)	0.01±0.01	0.03±0.01
sentiment	0.01±0.01	0.02±0.02
sentiment (NFT)	-	0.02±0.01
hhh	-	-
hhh (NFT)	-	-
freeman	-	0.02±0.01
freeman (NFT)	-	-
glados	-	-
glados (NFT)	-	-
german	-	-
german (NFT)	-	-

Table 28. Setup D' (comp. Table 9) with projective prompts.

Meta-Llama-3-8B-Instruct (1PP)		
Case	1H	2H
hhh	0.05±0.03	-
hhh (NFT)	0.35±0.10	-
freeman	-	-
freeman (NFT)	-	-
glados	-	-
glados (NFT)	0.04±0.02	-
german	-	-
german (NFT)	-	-

Mistral-7B-Instruct-v0.3 (1PP)		
Case	1H	2H
hhh	0.01±0.01	-
hhh (NFT)	0.01±0.01	-
freeman	-	-
freeman (NFT)	-	-
glados	-	-
glados (NFT)	-	-
german	-	-
german (NFT)	-	-

falcon-7b-instruct (1PP)		
Case	1H	2H
hhh	-	-
hhh (NFT)	-	-
freeman	-	-
freeman (NFT)	-	0.00±0.01
glados	-	-
glados (NFT)	-	-
german	-	-
german (NFT)	-	-

Meta-Llama-3-8B-Instruct (3PP)		
Case	1H	2H
hhh	0.60±0.04	0.03±0.01
hhh (NFT)	0.99±0.00	0.01±0.01
freeman	0.01±0.01	-
freeman (NFT)	0.03±0.02	0.00±0.01
glados	0.02±0.01	-
glados (NFT)	0.39±0.09	-
german	-	-
german (NFT)	-	-

Mistral-7B-Instruct-v0.3 (3PP)		
Case	1H	2H
hhh	0.49±0.13	-
hhh (NFT)	0.36±0.10	0.05±0.04
freeman	0.02±0.00	-
freeman (NFT)	0.08±0.04	-
glados	0.01±0.00	0.00±0.01
glados (NFT)	0.01±0.01	-
german	-	-
german (NFT)	0.02±0.01	-

falcon-7b-instruct (3PP)		
Case	1H	2H
hhh	-	-
hhh (NFT)	-	-
freeman	-	-
freeman (NFT)	-	-
glados	-	-
glados (NFT)	-	-
german	-	-
german (NFT)	-	-

Table 29. Setup D' (comp. Table 9) with associative prompts.

Meta-Llama-3-8B-Instruct (1PP)		
Case	1H	2H
hhh	0.13±0.04	0.01±0.01
hhh (NFT)	0.11±0.01	-
freeman	0.09±0.07	0.01±0.01
freeman (NFT)	0.06±0.03	0.01±0.01
glados	0.03±0.01	-
glados (NFT)	0.09±0.02	-
german	-	-
german (NFT)	-	-

Mistral-7B-Instruct-v0.3 (1PP)		
Case	1H	2H
hhh	-	-
hhh (NFT)	-	-
freeman	0.08±0.04	-
freeman (NFT)	0.51±0.08	-
glados	0.03±0.02	0.03±0.02
glados (NFT)	0.10±0.10	0.01±0.01
german	-	-
german (NFT)	-	-

falcon-7b-instruct (1PP)		
Case	1H	2H
hhh	-	-
hhh (NFT)	-	-
freeman	-	0.01±0.01
freeman (NFT)	-	-
glados	-	0.01±0.01
glados (NFT)	-	-
german	-	-
german (NFT)	-	-

Meta-Llama-3-8B-Instruct (3PP)		
Case	1H	2H
hhh	0.45±0.08	0.14±0.06
hhh (NFT)	0.64±0.20	0.07±0.03
freeman	0.02±0.02	0.01±0.01
freeman (NFT)	0.01±0.02	-
glados	0.01±0.01	-
glados (NFT)	0.05±0.02	0.02±0.01
german	-	-
german (NFT)	-	-

Mistral-7B-Instruct-v0.3 (3PP)		
Case	1H	2H
hhh	0.71±0.02	0.12±0.06
hhh (NFT)	0.73±0.04	0.02±0.01
freeman	0.16±0.03	0.01±0.01
freeman (NFT)	0.30±0.08	-
glados	0.10±0.02	-
glados (NFT)	0.02±0.02	-
german	-	-
german (NFT)	-	-

falcon-7b-instruct (3PP)		
Case	1H	2H
hhh	-	-
hhh (NFT)	-	-
freeman	-	-
freeman (NFT)	-	-
glados	-	-
glados (NFT)	-	-
german	-	-
german (NFT)	-	-

Table 30. Setup F (comp. Table 9) with standard prompts.

Meta-Llama-3-8B-Instruct (1PP)		
Case	1H	2H
calling	0.11±0.06	0.01±0.01
calling (NFT)	0.02±0.02	0.02±0.01
antonym	0.01±0.01	-
antonym (NFT)	-	-
name	-	-
name (NFT)	-	-
sentiment	0.01±0.01	-
sentiment (NFT)	-	0.01±0.01
hhh	0.01±0.01	-
hhh (NFT)	-	-
freeman	-	-
freeman (NFT)	-	-
glados	-	-
glados (NFT)	-	-
german	-	-
german (NFT)	-	-

Mistral-7B-Instruct-v0.3 (1PP)		
Case	1H	2H
calling	0.01±0.01	-
calling (NFT)	0.01±0.01	-
antonym	0.01±0.01	0.01±0.01
antonym (NFT)	0.01±0.01	0.01±0.01
name	-	-
name (NFT)	0.01±0.01	-
sentiment	0.01±0.01	-
sentiment (NFT)	-	-
hhh	-	-
hhh (NFT)	0.01±0.01	-
freeman	-	-
freeman (NFT)	-	-
glados	-	-
glados (NFT)	-	-
german	-	-
german (NFT)	-	-

falcon-7b-instruct (1PP)		
Case	1H	2H
calling	0.02±0.00	0.01±0.01
calling (NFT)	-	-
antonym	-	-
antonym (NFT)	-	-
name	0.01±0.01	-
name (NFT)	0.01±0.01	-
sentiment	-	0.02±0.01
sentiment (NFT)	-	-
hhh	-	-
hhh (NFT)	-	-
freeman	-	-
freeman (NFT)	-	-
glados	-	-
glados (NFT)	-	-
german	-	-
german (NFT)	-	-

Meta-Llama-3-8B-Instruct (3PP)		
Case	1H	2H
calling	0.13±0.08	-
calling (NFT)	0.39±0.25	0.01±0.01
antonym	0.07±0.05	0.02±0.01
antonym (NFT)	0.03±0.02	0.05±0.02
name	0.41±0.09	0.03±0.01
name (NFT)	0.05±0.02	0.08±0.01
sentiment	0.05±0.03	0.02±0.02
sentiment (NFT)	0.04±0.06	0.01±0.01
hhh	0.03±0.02	-
hhh (NFT)	-	-
freeman	-	-
freeman (NFT)	-	-
glados	-	-
glados (NFT)	-	-
german	-	-
german (NFT)	-	-

Mistral-7B-Instruct-v0.3 (3PP)		
Case	1H	2H
calling	0.24±0.02	-
calling (NFT)	0.15±0.04	-
antonym	0.97±0.02	0.01±0.01
antonym (NFT)	0.44±0.24	0.02±0.02
name	0.57±0.06	0.03±0.00
name (NFT)	0.41±0.01	0.02±0.01
sentiment	0.05±0.01	0.01±0.01
sentiment (NFT)	0.33±0.22	-
hhh	0.07±0.01	0.03±0.02
hhh (NFT)	0.06±0.03	0.06±0.01
freeman	-	-
freeman (NFT)	0.01±0.01	-
glados	-	-
glados (NFT)	-	-
german	-	-
german (NFT)	-	-

falcon-7b-instruct (3PP)		
Case	1H	2H
calling	0.01±0.01	-
calling (NFT)	0.01±0.02	-
antonym	0.01±0.01	-
antonym (NFT)	-	0.01±0.01
name	-	-
name (NFT)	-	-
sentiment	-	0.01±0.01
sentiment (NFT)	0.01±0.01	0.02±0.01
hhh	-	-
hhh (NFT)	-	-
freeman	-	-
freeman (NFT)	-	-
glados	-	-
glados (NFT)	-	-
german	-	-
german (NFT)	-	-

Table 31. Setup F (comp. Table 9) with projective prompts.

Meta-Llama-3-8B-Instruct (1PP)		
Case	1H	2H
hhh	0.04±0.04	-
hhh (NFT)	-	-
freeman	-	-
freeman (NFT)	-	-
glados	-	-
glados (NFT)	-	-
german	-	-
german (NFT)	-	-

Mistral-7B-Instruct-v0.3 (1PP)		
Case	1H	2H
hhh	0.05±0.06	-
hhh (NFT)	0.07±0.04	0.00±0.01
freeman	-	-
freeman (NFT)	-	-
glados	-	-
glados (NFT)	-	-
german	-	-
german (NFT)	-	-

falcon-7b-instruct (1PP)		
Case	1H	2H
hhh	-	-
hhh (NFT)	-	-
freeman	-	-
freeman (NFT)	-	-
glados	-	-
glados (NFT)	-	-
german	-	-
german (NFT)	-	-

Meta-Llama-3-8B-Instruct (3PP)		
Case	1H	2H
hhh	0.54±0.25	-
hhh (NFT)	0.04±0.03	0.00±0.01
freeman	-	-
freeman (NFT)	0.01±0.01	-
glados	-	0.00±0.01
glados (NFT)	-	-
german	-	-
german (NFT)	-	-

Mistral-7B-Instruct-v0.3 (3PP)		
Case	1H	2H
hhh	0.63±0.12	0.12±0.05
hhh (NFT)	0.78±0.09	0.20±0.05
freeman	-	-
freeman (NFT)	0.02±0.02	-
glados	0.01±0.01	-
glados (NFT)	-	-
german	-	-
german (NFT)	-	-

falcon-7b-instruct (3PP)		
Case	1H	2H
hhh	-	-
hhh (NFT)	-	-
freeman	-	-
freeman (NFT)	-	-
glados	-	-
glados (NFT)	-	-
german	-	-
german (NFT)	-	-

Table 32. Setup F (comp. Table 9) with associative prompts.

Meta-Llama-3-8B-Instruct (1PP)		
Case	1H	2H
hhh	0.02±0.03	-
hhh (NFT)	-	-
freeman	0.01±0.01	-
freeman (NFT)	0.02±0.02	-
glados	-	-
glados (NFT)	0.01±0.01	0.02±0.02
german	-	-
german (NFT)	-	-

Mistral-7B-Instruct-v0.3 (1PP)		
Case	1H	2H
hhh	-	-
hhh (NFT)	-	-
freeman	0.03±0.01	0.02±0.01
freeman (NFT)	0.21±0.07	0.02±0.04
glados	0.04±0.00	-
glados (NFT)	0.01±0.02	0.01±0.01
german	-	-
german (NFT)	-	-

falcon-7b-instruct (1PP)		
Case	1H	2H
hhh	-	-
hhh (NFT)	-	-
freeman	-	0.01±0.01
freeman (NFT)	-	-
glados	0.01±0.01	-
glados (NFT)	-	-
german	-	-
german (NFT)	-	-

Meta-Llama-3-8B-Instruct (3PP)		
Case	1H	2H
hhh	0.53±0.19	0.11±0.04
hhh (NFT)	0.41±0.02	0.07±0.03
freeman	0.03±0.02	0.01±0.01
freeman (NFT)	0.02±0.02	-
glados	0.01±0.01	0.01±0.01
glados (NFT)	0.02±0.00	-
german	-	-
german (NFT)	-	-

Mistral-7B-Instruct-v0.3 (3PP)		
Case	1H	2H
hhh	0.71±0.11	0.12±0.02
hhh (NFT)	0.65±0.06	0.07±0.02
freeman	0.02±0.02	-
freeman (NFT)	0.19±0.13	-
glados	0.12±0.06	-
glados (NFT)	0.17±0.03	0.01±0.01
german	-	-
german (NFT)	-	-

falcon-7b-instruct (3PP)		
Case	1H	2H
hhh	-	-
hhh (NFT)	-	-
freeman	-	-
freeman (NFT)	-	-
glados	-	-
glados (NFT)	-	-
german	-	-
german (NFT)	-	-

Table 33. Setup E (comp. Table 9) with standard prompts.

	Meta-Llama-3-8B-Instruct (1PP)		Mistral-7B-Instruct-v0.3 (1PP)		falcon-7b-instruct (1PP)	
Case	1H	2H	1H	2H	1H	2H
calling	-	-	-	-	-	-
calling (NFT)	-	-	-	-	-	-
antonym	-	0.01±0.01	-	0.01±0.01	-	-
antonym (NFT)	0.02±0.02	-	-	-	-	-
name	0.01±0.01	0.03±0.02	-	-	-	0.01±0.01
name (NFT)	-	0.02±0.02	-	-	0.01±0.01	-
sentiment	0.01±0.01	-	0.01±0.01	-	0.01±0.01	0.01±0.01
sentiment (NFT)	0.01±0.01	-	-	-	0.01±0.01	-
hhh	-	-	-	-	-	-
hhh (NFT)	-	-	-	-	-	-
freeman	-	-	-	-	-	-
freeman (NFT)	-	-	-	-	-	0.01±0.01
glados	-	-	-	-	-	-
glados (NFT)	-	-	-	-	-	-
german	-	-	-	-	-	-
german (NFT)	-	-	-	-	-	-

	Meta-Llama-3-8B-Instruct (3PP)		Mistral-7B-Instruct-v0.3 (3PP)		falcon-7b-instruct (3PP)	
Case	1H	2H	1H	2H	1H	2H
calling	0.02±0.00	0.01±0.01	-	-	-	-
calling (NFT)	0.01±0.01	0.01±0.01	-	-	-	-
antonym	0.02±0.02	0.02±0.01	0.03±0.02	0.02±0.02	0.01±0.01	-
antonym (NFT)	0.03±0.02	-	0.09±0.07	0.01±0.01	0.01±0.01	-
name	-	-	-	-	0.07±0.01	0.02±0.01
name (NFT)	0.02±0.00	-	0.01±0.01	-	0.04±0.00	0.03±0.00
sentiment	-	0.02±0.04	0.01±0.01	0.02±0.02	-	0.02±0.01
sentiment (NFT)	0.03±0.02	0.01±0.01	0.01±0.02	0.01±0.01	-	0.03±0.01
hhh	-	-	-	-	-	-
hhh (NFT)	-	-	-	-	-	-
freeman	0.01±0.01	0.01±0.01	-	-	-	0.01±0.01
freeman (NFT)	0.01±0.01	-	-	-	-	0.01±0.01
glados	0.01±0.01	-	-	-	-	-
glados (NFT)	-	-	-	-	-	-
german	-	-	-	-	-	-
german (NFT)	-	-	-	-	-	-

Table 34. Setup E (comp. Table 9) with projective prompts.

	Meta-Llama-3-8B-Instruct (1PP)		Mistral-7B-Instruct-v0.3 (1PP)		falcon-7b-instruct (1PP)	
Case	1H	2H	1H	2H	1H	2H
hhh	-	-	-	-	-	-
hhh (NFT)	-	-	-	-	-	-
freeman	-	-	-	-	-	-
freeman (NFT)	-	-	-	-	-	-
glados	-	-	-	-	-	-
glados (NFT)	-	-	-	-	-	-
german	-	-	-	-	-	-
german (NFT)	-	-	-	-	-	-

	Meta-Llama-3-8B-Instruct (3PP)		Mistral-7B-Instruct-v0.3 (3PP)		falcon-7b-instruct (3PP)	
Case	1H	2H	1H	2H	1H	2H
hhh	-	-	-	-	-	-
hhh (NFT)	-	-	-	-	-	-
freeman	-	0.00±0.01	-	-	-	-
freeman (NFT)	-	-	-	-	-	-
glados	-	0.00±0.01	-	0.00±0.01	-	-
glados (NFT)	-	-	-	-	-	-
german	-	-	-	-	-	-
german (NFT)	-	-	-	-	-	-

Table 35. Setup E (comp. Table 9) with associative prompts.

	Meta-Llama-3-8B-Instruct (1PP)		Mistral-7B-Instruct-v0.3 (1PP)		falcon-7b-instruct (1PP)	
Case	1H	2H	1H	2H	1H	2H
hhh	-	-	-	-	-	-
hhh (NFT)	-	-	-	-	-	-
freeman	0.02±0.02	0.02±0.01	0.01±0.01	0.01±0.01	-	-
freeman (NFT)	0.03±0.01	0.01±0.01	0.01±0.01	-	-	-
glados	0.01±0.01	-	-	0.01±0.01	-	-
glados (NFT)	0.01±0.01	-	0.01±0.01	0.01±0.01	-	-
german	-	-	-	-	-	-
german (NFT)	-	-	-	-	-	-

	Meta-Llama-3-8B-Instruct (3PP)		Mistral-7B-Instruct-v0.3 (3PP)		falcon-7b-instruct (3PP)	
Case	1H	2H	1H	2H	1H	2H
hhh	-	-	-	-	-	-
hhh (NFT)	-	-	-	-	-	-
freeman	-	-	0.01±0.01	-	-	-
freeman (NFT)	-	-	0.03±0.01	0.02±0.01	-	-
glados	-	-	-	-	-	-
glados (NFT)	-	-	0.01±0.01	-	-	-
german	-	-	-	-	-	-
german (NFT)	-	-	0.01±0.02	-	-	-

Table 36. Setup G (comp. Table 9) with standard prompts.

Meta-Llama-3-8B-Instruct (1PP)		
Case	1H	2H
calling	-	-
calling (NFT)	-	-
antonym	-	-
antonym (NFT)	-	-
name	-	-
name (NFT)	-	-
sentiment	-	-
sentiment (NFT)	-	-
hhh	-	-
hhh (NFT)	-	-
freeman	-	-
freeman (NFT)	-	-
glados	-	-
glados (NFT)	-	-
german	-	-
german (NFT)	-	-

Mistral-7B-Instruct-v0.3 (1PP)		
Case	1H	2H
calling	-	-
calling (NFT)	-	-
antonym	-	-
antonym (NFT)	-	0.01±0.01
name	-	-
name (NFT)	-	-
sentiment	-	-
sentiment (NFT)	-	-
hhh	-	-
hhh (NFT)	-	-
freeman	-	-
freeman (NFT)	-	-
glados	-	-
glados (NFT)	-	-
german	-	-
german (NFT)	-	-

falcon-7b-instruct (1PP)		
Case	1H	2H
calling	0.01±0.01	-
calling (NFT)	0.01±0.01	0.02±0.02
antonym	-	-
antonym (NFT)	0.01±0.01	-
name	0.01±0.01	0.01±0.01
name (NFT)	0.01±0.01	0.04±0.01
sentiment	-	0.01±0.01
sentiment (NFT)	-	-
hhh	-	-
hhh (NFT)	-	-
freeman	-	0.01±0.01
freeman (NFT)	-	-
glados	-	-
glados (NFT)	-	-
german	-	-
german (NFT)	0.01±0.01	-

Meta-Llama-3-8B-Instruct (3PP)		
Case	1H	2H
calling	-	-
calling (NFT)	-	-
antonym	0.07±0.04	0.01±0.01
antonym (NFT)	0.01±0.01	0.02±0.01
name	-	-
name (NFT)	-	0.01±0.01
sentiment	0.01±0.01	-
sentiment (NFT)	-	0.01±0.01
hhh	-	-
hhh (NFT)	-	-
freeman	0.01±0.01	-
freeman (NFT)	-	-
glados	0.01±0.02	-
glados (NFT)	-	-
german	-	-
german (NFT)	-	-

Mistral-7B-Instruct-v0.3 (3PP)		
Case	1H	2H
calling	-	-
calling (NFT)	-	-
antonym	0.01±0.01	0.01±0.01
antonym (NFT)	0.08±0.06	0.01±0.01
name	-	-
name (NFT)	-	-
sentiment	0.01±0.01	0.01±0.01
sentiment (NFT)	0.01±0.02	-
hhh	-	-
hhh (NFT)	-	-
freeman	0.01±0.01	-
freeman (NFT)	-	-
glados	-	-
glados (NFT)	-	-
german	-	-
german (NFT)	-	-

falcon-7b-instruct (3PP)		
Case	1H	2H
calling	-	-
calling (NFT)	0.02±0.03	-
antonym	0.02±0.02	0.01±0.01
antonym (NFT)	0.01±0.02	-
name	-	0.01±0.01
name (NFT)	0.01±0.01	0.01±0.01
sentiment	-	0.03±0.02
sentiment (NFT)	-	0.03±0.03
hhh	-	-
hhh (NFT)	-	-
freeman	0.01±0.01	-
freeman (NFT)	-	0.01±0.01
glados	-	-
glados (NFT)	-	-
german	-	-
german (NFT)	-	-

Table 37. Setup G (comp. Table 9) with projective prompts.

Meta-Llama-3-8B-Instruct (1PP)		
Case	1H	2H
hhh	-	-
hhh (NFT)	-	-
freeman	-	-
freeman (NFT)	-	-
glados	-	-
glados (NFT)	-	-
german	-	-
german (NFT)	-	-

Mistral-7B-Instruct-v0.3 (1PP)		
Case	1H	2H
hhh	-	-
hhh (NFT)	-	-
freeman	-	-
freeman (NFT)	-	-
glados	-	-
glados (NFT)	-	-
german	-	-
german (NFT)	-	-

falcon-7b-instruct (1PP)		
Case	1H	2H
hhh	-	-
hhh (NFT)	-	-
freeman	-	-
freeman (NFT)	-	-
glados	-	-
glados (NFT)	-	-
german	-	-
german (NFT)	-	-

Meta-Llama-3-8B-Instruct (3PP)		
Case	1H	2H
hhh	-	-
hhh (NFT)	-	-
freeman	-	-
freeman (NFT)	-	-
glados	-	-
glados (NFT)	-	-
german	-	-
german (NFT)	-	-

Mistral-7B-Instruct-v0.3 (3PP)		
Case	1H	2H
hhh	-	-
hhh (NFT)	-	-
freeman	-	-
freeman (NFT)	0.01±0.00	-
glados	-	-
glados (NFT)	-	-
german	-	0.00±0.01
german (NFT)	-	-

falcon-7b-instruct (3PP)		
Case	1H	2H
hhh	-	-
hhh (NFT)	-	-
freeman	-	0.00±0.01
freeman (NFT)	-	-
glados	-	-
glados (NFT)	-	-
german	-	-
german (NFT)	-	-

Table 38. Setup G (comp. Table 9) with associative prompts.

Meta-Llama-3-8B-Instruct (1PP)		
Case	1H	2H
hhh	-	-
hhh (NFT)	-	-
freeman	0.02±0.03	0.02±0.02
freeman (NFT)	-	-
glados	0.01±0.01	0.01±0.01
glados (NFT)	-	-
german	-	-
german (NFT)	-	-

Mistral-7B-Instruct-v0.3 (1PP)		
Case	1H	2H
hhh	-	-
hhh (NFT)	-	-
freeman	0.01±0.01	0.01±0.01
freeman (NFT)	0.03±0.02	0.01±0.01
glados	-	-
glados (NFT)	-	-
german	-	-
german (NFT)	-	-

falcon-7b-instruct (1PP)		
Case	1H	2H
hhh	-	-
hhh (NFT)	-	-
freeman	0.01±0.01	0.01±0.01
freeman (NFT)	-	-
glados	-	-
glados (NFT)	-	-
german	-	-
german (NFT)	-	-

Meta-Llama-3-8B-Instruct (3PP)		
Case	1H	2H
hhh	-	-
hhh (NFT)	-	-
freeman	-	0.01±0.01
freeman (NFT)	-	-
glados	-	-
glados (NFT)	-	-
german	-	-
german (NFT)	-	-

Mistral-7B-Instruct-v0.3 (3PP)		
Case	1H	2H
hhh	-	-
hhh (NFT)	-	-
freeman	0.01±0.01	-
freeman (NFT)	-	0.01±0.01
glados	-	-
glados (NFT)	-	0.01±0.01
german	-	-
german (NFT)	-	-

falcon-7b-instruct (3PP)		
Case	1H	2H
hhh	-	-
hhh (NFT)	-	-
freeman	0.01±0.01	-
freeman (NFT)	-	-
glados	-	-
glados (NFT)	-	-
german	-	-
german (NFT)	0.01±0.01	-

Table 39. Setup H (comp. Table 9) with standard prompts.

Meta-Llama-3-8B-Instruct (1PP)		
Case	1H	2H
calling	-	-
calling (NFT)	-	0.03$\pm$0.00
antonym	-	-
antonym (NFT)	-	-
name	-	-
name (NFT)	-	-
sentiment	-	-
sentiment (NFT)	-	-
hhh	-	-
hhh (NFT)	-	-
freeman	-	-
freeman (NFT)	-	-
glados	-	-
glados (NFT)	-	-
german	-	-
german (NFT)	-	-

Mistral-7B-Instruct-v0.3 (1PP)		
Case	1H	2H
calling	-	-
calling (NFT)	-	0.03$\pm$0.00
antonym	-	-
antonym (NFT)	-	-
name	-	-
name (NFT)	-	-
sentiment	-	-
sentiment (NFT)	-	0.05$\pm$0.00
hhh	-	-
hhh (NFT)	-	-
freeman	-	-
freeman (NFT)	-	-
glados	-	-
glados (NFT)	-	-
german	-	-
german (NFT)	-	-

falcon-7b-instruct (1PP)		
Case	1H	2H
calling	-	-
calling (NFT)	-	-
antonym	-	-
antonym (NFT)	-	-
name	-	-
name (NFT)	-	-
sentiment	-	-
sentiment (NFT)	-	-
hhh	-	-
hhh (NFT)	-	-
freeman	-	-
freeman (NFT)	-	-
glados	-	-
glados (NFT)	-	-
german	-	0.03$\pm$0.00
german (NFT)	-	-

Meta-Llama-3-8B-Instruct (3PP)		
Case	1H	2H
calling	-	-
calling (NFT)	-	-
antonym	-	-
antonym (NFT)	0.01$\pm$0.01	0.02$\pm$0.01
name	-	-
name (NFT)	0.02$\pm$0.00	-
sentiment	-	-
sentiment (NFT)	-	0.03$\pm$0.00
hhh	-	-
hhh (NFT)	-	-
freeman	-	0.03$\pm$0.00
freeman (NFT)	-	-
glados	-	-
glados (NFT)	-	-
german	-	-
german (NFT)	-	-

Mistral-7B-Instruct-v0.3 (3PP)		
Case	1H	2H
calling	0.04$\pm$0.00	-
calling (NFT)	-	-
antonym	0.02$\pm$0.00	0.03$\pm$0.00
antonym (NFT)	0.02$\pm$0.00	-
name	-	-
name (NFT)	-	-
sentiment	0.04$\pm$0.00	0.07$\pm$0.00
sentiment (NFT)	-	0.05$\pm$0.00
hhh	-	-
hhh (NFT)	-	-
freeman	0.02$\pm$0.00	0.03$\pm$0.00
freeman (NFT)	0.02$\pm$0.00	-
glados	-	-
glados (NFT)	-	-
german	-	-
german (NFT)	-	-

falcon-7b-instruct (3PP)		
Case	1H	2H
calling	-	-
calling (NFT)	-	-
antonym	-	-
antonym (NFT)	-	-
name	-	-
name (NFT)	-	-
sentiment	-	-
sentiment (NFT)	-	-
hhh	-	-
hhh (NFT)	-	-
freeman	-	-
freeman (NFT)	-	-
glados	-	-
glados (NFT)	-	-
german	-	-
german (NFT)	-	-

Table 40. Setup H (comp. Table 9) with projective prompts.

Meta-Llama-3-8B-Instruct (1PP)		
Case	1H	2H
hhh	-	-
hhh (NFT)	-	-
freeman	-	-
freeman (NFT)	-	-
glados	-	-
glados (NFT)	-	-
german	-	-
german (NFT)	-	-

Mistral-7B-Instruct-v0.3 (1PP)		
Case	1H	2H
hhh	-	-
hhh (NFT)	-	-
freeman	-	-
freeman (NFT)	-	-
glados	-	-
glados (NFT)	-	-
german	-	-
german (NFT)	-	-

falcon-7b-instruct (1PP)		
Case	1H	2H
hhh	-	-
hhh (NFT)	-	-
freeman	-	-
freeman (NFT)	-	-
glados	-	-
glados (NFT)	-	-
german	-	-
german (NFT)	0.01$\pm$0.00	-

Meta-Llama-3-8B-Instruct (3PP)		
Case	1H	2H
hhh	-	-
hhh (NFT)	-	-
freeman	-	-
freeman (NFT)	-	-
glados	0.01$\pm$0.00	-
glados (NFT)	-	-
german	-	-
german (NFT)	-	-

Mistral-7B-Instruct-v0.3 (3PP)		
Case	1H	2H
hhh	-	-
hhh (NFT)	-	-
freeman	-	0.01$\pm$0.00
freeman (NFT)	0.01$\pm$0.00	0.01$\pm$0.00
glados	-	-
glados (NFT)	0.01$\pm$0.00	0.01$\pm$0.00
german	-	-
german (NFT)	-	-

falcon-7b-instruct (3PP)		
Case	1H	2H
hhh	-	-
hhh (NFT)	-	-
freeman	-	-
freeman (NFT)	-	-
glados	-	-
glados (NFT)	-	-
german	-	0.02$\pm$0.01
german (NFT)	-	0.03$\pm$0.01

Table 41. Setup H (comp. Table 9) with associative prompts.

Meta-Llama-3-8B-Instruct (1PP)		
Case	1H	2H
hhh	-	-
hhh (NFT)	-	-
freeman	-	0.03$\pm$0.00
freeman (NFT)	-	-
glados	-	-
glados (NFT)	-	-
german	-	-
german (NFT)	-	-

Mistral-7B-Instruct-v0.3 (1PP)		
Case	1H	2H
hhh	-	-
hhh (NFT)	-	-
freeman	-	-
freeman (NFT)	0.02$\pm$0.00	0.03$\pm$0.00
glados	-	-
glados (NFT)	-	-
german	-	-
german (NFT)	-	-

falcon-7b-instruct (1PP)		
Case	1H	2H
hhh	-	-
hhh (NFT)	-	-
freeman	-	-
freeman (NFT)	-	-
glados	-	-
glados (NFT)	-	-
german	0.01$\pm$0.01	-
german (NFT)	-	-

Meta-Llama-3-8B-Instruct (3PP)		
Case	1H	2H
hhh	-	-
hhh (NFT)	-	-
freeman	-	-
freeman (NFT)	-	-
glados	-	-
glados (NFT)	-	-
german	-	-
german (NFT)	-	-

Mistral-7B-Instruct-v0.3 (3PP)		
Case	1H	2H
hhh	-	-
hhh (NFT)	-	-
freeman	-	-
freeman (NFT)	0.02$\pm$0.00	-
glados	-	-
glados (NFT)	-	-
german	-	-
german (NFT)	-	-

falcon-7b-instruct (3PP)		
Case	1H	2H
hhh	-	-
hhh (NFT)	-	-
freeman	-	-
freeman (NFT)	-	-
glados	-	-
glados (NFT)	-	-
german	0.01$\pm$0.01	-
german (NFT)	0.02$\pm$0.00	0.01$\pm$0.01

Table 42. Setup I (comp. Table 9) with standard prompts.

Meta-Llama-3-8B-Instruct (1PP)		
Case	1H	2H
calling	-	-
calling (NFT)	-	-
antonym	-	-
antonym (NFT)	0.01 ± 0.01	-
name	-	-
name (NFT)	-	-
sentiment	-	-
sentiment (NFT)	-	-
hhh	-	-
hhh (NFT)	-	-
freeman	-	-
freeman (NFT)	0.02 ± 0.00	0.03 ± 0.00
glados	-	-
glados (NFT)	-	-
german	-	-
german (NFT)	-	-

Mistral-7B-Instruct-v0.3 (1PP)		
Case	1H	2H
calling	-	-
calling (NFT)	-	-
antonym	-	-
antonym (NFT)	-	-
name	-	-
name (NFT)	-	-
sentiment	-	0.03 ± 0.00
sentiment (NFT)	-	-
hhh	-	-
hhh (NFT)	-	-
freeman	0.02 ± 0.00	-
freeman (NFT)	0.02 ± 0.00	0.03 ± 0.00
glados	-	-
glados (NFT)	-	-
german	-	-
german (NFT)	-	-

falcon-7b-instruct (1PP)		
Case	1H	2H
calling	-	-
calling (NFT)	-	-
antonym	-	-
antonym (NFT)	-	-
name	-	-
name (NFT)	-	-
sentiment	-	-
sentiment (NFT)	-	-
hhh	-	-
hhh (NFT)	-	-
freeman	-	-
freeman (NFT)	-	-
glados	-	-
glados (NFT)	-	-
german	-	-
german (NFT)	-	-

Meta-Llama-3-8B-Instruct (3PP)		
Case	1H	2H
calling	-	-
calling (NFT)	-	-
antonym	0.02 ± 0.00	-
antonym (NFT)	-	-
name	0.02 ± 0.00	0.05 ± 0.00
name (NFT)	0.02 ± 0.00	0.05 ± 0.00
sentiment	-	-
sentiment (NFT)	-	-
hhh	-	-
hhh (NFT)	-	-
freeman	-	-
freeman (NFT)	-	-
glados	-	-
glados (NFT)	-	-
german	-	-
german (NFT)	-	-

Mistral-7B-Instruct-v0.3 (3PP)		
Case	1H	2H
calling	-	-
calling (NFT)	-	-
antonym	-	0.03 ± 0.00
antonym (NFT)	-	0.01 ± 0.01
name	-	0.03 ± 0.00
name (NFT)	-	0.03 ± 0.00
sentiment	0.02 ± 0.00	-
sentiment (NFT)	-	-
hhh	-	-
hhh (NFT)	-	-
freeman	-	0.03 ± 0.00
freeman (NFT)	0.02 ± 0.00	-
glados	-	-
glados (NFT)	-	-
german	-	-
german (NFT)	-	-

falcon-7b-instruct (3PP)		
Case	1H	2H
calling	-	-
calling (NFT)	-	-
antonym	-	-
antonym (NFT)	-	-
name	-	-
name (NFT)	-	-
sentiment	-	-
sentiment (NFT)	-	-
hhh	-	-
hhh (NFT)	-	-
freeman	-	-
freeman (NFT)	-	-
glados	-	-
glados (NFT)	-	-
german	-	-
german (NFT)	-	-

Table 43. Setup I (comp. Table 9) with projective prompts.

Meta-Llama-3-8B-Instruct (1PP)		
Case	1H	2H
hhh	-	-
hhh (NFT)	-	-
freeman	0.01 ± 0.00	-
freeman (NFT)	0.04 ± 0.00	-
glados	0.01 ± 0.00	-
glados (NFT)	-	-
german	0.01 ± 0.00	0.00 ± 0.01
german (NFT)	-	-

Mistral-7B-Instruct-v0.3 (1PP)		
Case	1H	2H
hhh	-	-
hhh (NFT)	-	-
freeman	-	-
freeman (NFT)	0.01 ± 0.00	-
glados	-	-
glados (NFT)	-	-
german	-	-
german (NFT)	-	-

falcon-7b-instruct (1PP)		
Case	1H	2H
hhh	-	-
hhh (NFT)	-	-
freeman	-	-
freeman (NFT)	-	-
glados	-	-
glados (NFT)	-	-
german	-	-
german (NFT)	-	-

Meta-Llama-3-8B-Instruct (3PP)		
Case	1H	2H
hhh	-	-
hhh (NFT)	-	-
freeman	-	-
freeman (NFT)	-	-
glados	-	-
glados (NFT)	-	-
german	-	-
german (NFT)	0.01 ± 0.00	-

Mistral-7B-Instruct-v0.3 (3PP)		
Case	1H	2H
hhh	-	-
hhh (NFT)	-	-
freeman	-	-
freeman (NFT)	0.01 ± 0.00	-
glados	-	-
glados (NFT)	-	-
german	-	-
german (NFT)	-	-

falcon-7b-instruct (3PP)		
hhh	-	-
hhh (NFT)	-	-
freeman	-	-
freeman (NFT)	-	-
glados	-	-
glados (NFT)	-	-
german	-	-
german (NFT)	-	-

Table 44. Setup I (comp. Table 9) with associative prompts.

Meta-Llama-3-8B-Instruct (1PP)		
Case	1H	2H
hhh	-	-
hhh (NFT)	-	-
freeman	-	-
freeman (NFT)	-	-
glados	-	-
glados (NFT)	-	-
german	-	-
german (NFT)	0.01 ± 0.01	-

Mistral-7B-Instruct-v0.3 (1PP)		
Case	1H	2H
hhh	-	-
hhh (NFT)	-	-
freeman	-	-
freeman (NFT)	-	-
glados	-	-
glados (NFT)	-	-
german	-	-
german (NFT)	-	-

falcon-7b-instruct (1PP)		
Case	1H	2H
hhh	-	-
hhh (NFT)	-	-
freeman	-	-
freeman (NFT)	-	-
glados	-	-
glados (NFT)	-	-
german	-	-
german (NFT)	-	-

Meta-Llama-3-8B-Instruct (3PP)		
Case	1H	2H
hhh	-	-
hhh (NFT)	-	-
freeman	-	-
freeman (NFT)	-	-
glados	-	-
glados (NFT)	-	-
german	-	-
german (NFT)	-	-

Mistral-7B-Instruct-v0.3 (3PP)		
Case	1H	2H
hhh	-	-
hhh (NFT)	-	-
freeman	-	-
freeman (NFT)	-	-
glados	-	-
glados (NFT)	-	-
german	-	-
german (NFT)	-	-

falcon-7b-instruct (3PP)		
Case	1H	2H
hhh	-	-
hhh (NFT)	-	-
freeman	-	-
freeman (NFT)	-	-
glados	-	-
glados (NFT)	-	-
german	-	-
german (NFT)	-	-

Table 45. Setup J (comp. Table 9) with standard prompts.

Meta-Llama-3.3-70B-Instruct (1:249) (1PP)		
Case	1H	2H
calling	-	-
calling (NFT)	-	-
antonym	0.02±0.00	-
antonym (NFT)	0.02±0.00	-
name	-	-
name (NFT)	-	-
sentiment	0.02±0.00	0.05±0.00
sentiment (NFT)	0.02±0.00	0.05±0.00
hhh	0.04±0.00	-
hhh (NFT)	0.04±0.00	-
freeman	-	-
freeman (NFT)	-	-
glados	-	-
glados (NFT)	-	-
german	-	-
german (NFT)	-	-

Meta-Llama-3.3-70B-Instruct (1:99) (1PP)		
Case	1H	2H
calling	-	-
calling (NFT)	-	-
antonym	0.02±0.00	-
antonym (NFT)	0.02±0.00	-
name	-	-
name (NFT)	-	-
sentiment	-	-
sentiment (NFT)	-	-
hhh	0.02±0.00	-
hhh (NFT)	-	-
freeman	-	-
freeman (NFT)	-	-
glados	-	-
glados (NFT)	-	-
german	-	-
german (NFT)	-	-

Meta-Llama-3.3-70B-Instruct (1:49) (1PP)		
Case	1H	2H
calling	-	-
calling (NFT)	-	-
antonym	-	-
antonym (NFT)	0.02±0.00	-
name	-	-
name (NFT)	-	-
sentiment	-	-
sentiment (NFT)	-	-
hhh	-	-
hhh (NFT)	-	-
freeman	-	-
freeman (NFT)	-	-
glados	-	-
glados (NFT)	-	-
german	-	-
german (NFT)	-	-

Meta-Llama-3.3-70B-Instruct (1:249) (3PP)		
Case	1H	2H
calling	0.26±0.00	-
calling (NFT)	0.20±0.00	-
antonym	1.00±0.00	0.03±0.00
antonym (NFT)	0.90±0.00	0.03±0.00
name	-	-
name (NFT)	0.02±0.00	-
sentiment	0.26±0.00	-
sentiment (NFT)	0.10±0.00	-
hhh	0.02±0.00	-
hhh (NFT)	0.04±0.00	-
freeman	-	-
freeman (NFT)	-	-
glados	-	-
glados (NFT)	-	-
german	-	-
german (NFT)	-	-

Meta-Llama-3.3-70B-Instruct (1:99) (3PP)		
Case	1H	2H
calling	0.06±0.00	-
calling (NFT)	0.10±0.00	-
antonym	1.00±0.00	0.05±0.00
antonym (NFT)	0.82±0.00	0.03±0.00
name	0.02±0.00	-
name (NFT)	0.02±0.00	-
sentiment	0.10±0.00	-
sentiment (NFT)	0.10±0.00	-
hhh	-	-
hhh (NFT)	-	-
freeman	-	-
freeman (NFT)	-	-
glados	-	-
glados (NFT)	-	-
german	-	-
german (NFT)	-	-

Meta-Llama-3.3-70B-Instruct (1:49) (3PP)		
Case	1H	2H
calling	0.08±0.00	-
calling (NFT)	0.02±0.00	-
antonym	0.98±0.00	0.10±0.00
antonym (NFT)	0.88±0.00	0.03±0.00
name	0.02±0.00	-
name (NFT)	0.02±0.00	-
sentiment	0.10±0.00	0.03±0.00
sentiment (NFT)	0.08±0.00	-
hhh	-	-
hhh (NFT)	-	-
freeman	-	-
freeman (NFT)	-	-
glados	-	-
glados (NFT)	-	-
german	-	-
german (NFT)	-	-

Table 46. Setup J (comp. Table 9) with projective prompts.

Meta-Llama-3.3-70B-Instruct (1:249) (1PP)		
Case	1H	2H
hhh	0.45±0.00	0.04±0.00
hhh (NFT)	0.04±0.00	0.01±0.00
freeman	-	-
freeman (NFT)	-	-
glados	0.01±0.00	-
glados (NFT)	-	-
german	-	-
german (NFT)	-	-

Meta-Llama-3.3-70B-Instruct (1:99) (1PP)		
Case	1H	2H
hhh	0.11±0.00	-
hhh (NFT)	0.01±0.00	-
freeman	-	-
freeman (NFT)	-	-
glados	0.02±0.00	-
glados (NFT)	-	-
german	-	-
german (NFT)	0.01±0.00	-

Meta-Llama-3.3-70B-Instruct (1:49) (1PP)		
Case	1H	2H
hhh	-	-
hhh (NFT)	-	-
freeman	-	-
freeman (NFT)	-	-
glados	0.01±0.00	-
glados (NFT)	-	-
german	-	-
german (NFT)	-	-

Meta-Llama-3.3-70B-Instruct (1:249) (3PP)		
Case	1H	2H
hhh	0.35±0.00	-
hhh (NFT)	0.02±0.00	-
freeman	-	-
freeman (NFT)	-	-
glados	0.04±0.00	-
glados (NFT)	-	-
german	0.01±0.00	-
german (NFT)	0.05±0.00	-

Meta-Llama-3.3-70B-Instruct (1:99) (3PP)		
Case	1H	2H
hhh	0.06±0.00	-
hhh (NFT)	0.01±0.00	-
freeman	-	-
freeman (NFT)	-	-
glados	0.02±0.00	-
glados (NFT)	0.05±0.00	-
german	-	-
german (NFT)	0.13±0.00	-

Meta-Llama-3.3-70B-Instruct (1:49) (3PP)		
Case	1H	2H
hhh	0.01±0.00	-
hhh (NFT)	0.02±0.00	-
freeman	-	-
freeman (NFT)	-	-
glados	0.02±0.00	0.01±0.00
glados (NFT)	0.05±0.00	-
german	-	-
german (NFT)	0.04±0.00	-

Table 47. Setup J (comp. Table 9) with associative prompts.

Meta-Llama-3.3-70B-Instruct (1:249) (1PP)		
Case	1H	2H
hhh	0.02±0.00	-
hhh (NFT)	-	-
freeman	-	0.03±0.00
freeman (NFT)	-	-
glados	0.02±0.00	-
glados (NFT)	-	-
german	-	-
german (NFT)	-	-

Meta-Llama-3.3-70B-Instruct (1:99) (1PP)		
Case	1H	2H
hhh	-	-
hhh (NFT)	0.02±0.00	-
freeman	-	-
freeman (NFT)	-	0.03±0.00
glados	0.04±0.00	-
glados (NFT)	0.02±0.00	-
german	-	-
german (NFT)	-	-

Meta-Llama-3.3-70B-Instruct (1:49) (1PP)		
Case	1H	2H
hhh	-	-
hhh (NFT)	0.02±0.00	-
freeman	-	-
freeman (NFT)	-	-
glados	-	-
glados (NFT)	0.02±0.00	-
german	-	-
german (NFT)	-	-

Meta-Llama-3.3-70B-Instruct (1:249) (3PP)		
Case	1H	2H
hhh	0.46±0.00	0.07±0.00
hhh (NFT)	0.42±0.00	0.60±0.00
freeman	0.04±0.00	-
freeman (NFT)	0.06±0.00	-
glados	0.08±0.00	0.03±0.00
glados (NFT)	-	-
german	-	-
german (NFT)	-	-

Meta-Llama-3.3-70B-Instruct (1:99) (3PP)		
Case	1H	2H
hhh	0.10±0.00	0.10±0.00
hhh (NFT)	0.42±0.00	0.12±0.00
freeman	0.04±0.00	-
freeman (NFT)	-	-
glados	0.10±0.00	0.03±0.00
glados (NFT)	-	0.03±0.00
german	-	-
german (NFT)	-	-

Meta-Llama-3.3-70B-Instruct (1:49) (3PP)		
Case	1H	2H
hhh	0.06±0.00	0.20±0.00
hhh (NFT)	0.48±0.00	0.28±0.00
freeman	-	-
freeman (NFT)	0.02±0.00	-
glados	0.06±0.00	0.03±0.00
glados (NFT)	0.04±0.00	-
german	-	-
german (NFT)	-	-

Table 48. Confidence intervals for our main experimental study when using first-person perspective (1PP) prompts.

Strategy →	1PP-STD		Strategy →	1PP-STD		1PP-PRO		1PP-ASS	
Number, Format →	N=50, in-temp.		Number, Format →	N=50, in-temp.		N=100, in-temp.		N=50, ex-temp.	
Case ↓ / Model →	Llama-3	Mistral	Case ↓ / Model →	Llama-3	Mistral	Llama-3	Mistral	Llama-3	Mistral
calling	[0.60, 0.68]	[0.07, 0.11]	hhh	[0.06, 0.10]	-	[0.36, 0.42]	[0.01, 0.03]	[0.15, 0.24]	[0.00, 0.02]
calling (NFT)	[0.03, 0.07]	[0.26, 0.33]	hhh	[0.19, 0.26]	-	[0.35, 0.41]	[0.01, 0.03]	[0.25, 0.35]	[0.00, 0.02]
antonym	-	-	freeman	-	-	-	-	[0.00, 0.03]	[0.00, 0.02]
antonym (NFT)	[0.03, 0.06]	[0.02, 0.04]	freeman (NFT)	-	-	-	-	[0.06, 0.12]	[0.03, 0.08]
name	-	-	glados	-	-	[0.00, 0.01]	-	[0.06, 0.13]	[0.02, 0.06]
name (NFT)	-	-	glados (NFT)	-	-	[0.00, 0.01]	-	[0.03, 0.08]	[0.00, 0.03]
sentiment	-	[0.00, 0.01]	german	-	-	-	-	[0.00, 0.02]	-
sentiment (NFT)	-	[0.00, 0.02]	german (NFT)	-	-	-	-	-	-

Confidence intervals based on the values in Table 1 for the response rate means, which we bootstrapped as explained in the "Uncertainty estimation" section for the standard (STD), projective (PRO) and associative (ASS) first-person (1PP) perspective prompts (dashes "-" substitute "[0.00, 0.00]"). "(NFT)" indicates that non-factorable tokens were used during fine-tuning and prompting, while "(in-temp.)" and "(ex-temp.)" show whether the prompts were embedded in the chat template or not. Values in columns 2 and 3 show the Llama-3 and Mistral results for the input-dependent cases (*calling*, *antonym*, *name*, *sentiment*), while the values in columns 5-10 show the corresponding results for the input-independent cases (*hhh*, *freeman*, *glados*, *german*). The gray background colour indicates that the *hhh* case is not covered by our definition of out-of-context reasoning and acts solely as a comparison/baseline.

Table 49. Confidence intervals for our main experimental study when using third-person perspective (3PP) prompts.

Strategy →	3PP-STD		Strategy →	3PP-STD		3PP-PRO		3PP-ASS	
Number, Format →	N=50, ex-temp.		Number, Format →	N=50, ex-temp.		N=100, ex-temp.		N=50, ex-temp.	
Case ↓ / Model →	Llama-3	Mistral	Case ↓ / Model →	Llama-3	Mistral	Llama-3	Mistral	Llama-3	Mistral
calling	[0.77, 0.83]	[0.47, 0.55]	hhh	[0.28, 0.36]	[0.00, 0.01]	[0.37, 0.42]	[0.63, 0.69]	[0.52, 0.63]	[0.63, 0.73]
calling (NFT)	[0.28, 0.35]	[0.50, 0.58]	hhh	[0.29, 0.37]	[0.01, 0.04]	[0.61, 0.67]	[0.47, 0.53]	[0.65, 0.75]	[0.62, 0.72]
antonym	[0.00, 0.02]	[0.54, 0.61]	freeman	-	[0.00, 0.01]	-	-	-	[0.02, 0.06]
antonym (NFT)	[0.11, 0.17]	[0.63, 0.70]	freeman (NFT)	-	[0.00, 0.01]	-	[0.00, 0.01]	[0.00, 0.03]	[0.07, 0.15]
name	[0.00, 0.02]	[0.47, 0.52]	glados	[0.00, 0.01]	-	[0.03, 0.05]	[0.00, 0.01]	[0.02, 0.06]	[0.07, 0.14]
name (NFT)	[0.03, 0.07]	[0.21, 0.28]	glados (NFT)	[0.00, 0.01]	-	[0.04, 0.06]	-	[0.04, 0.10]	-
sentiment	[0.00, 0.01]	[0.07, 0.11]	german	-	-	[0.00, 0.01]	[0.00, 0.01]	-	-
sentiment (NFT)	[0.20, 0.27]	[0.19, 0.26]	german (NFT)	-	[0.00, 0.02]	-	[0.27, 0.33]	-	[0.07, 0.13]

Confidence intervals based on the values in Table 2. Notation and layout as in Table 48.

Table 50. Confidence intervals for our main experimental study when using first-person perspective (1PP) prompts while exchanging the assistant names (single character difference).

Strategy →	1PP-STD		Strategy →	1PP-STD		1PP-PRO		1PP-ASS	
Number, Format →	N=50, in-temp.		Number, Format →	N=50, in-temp.		N=100, in-temp.		N=50, ex-temp.	
Case ↓ / Model →	Llama-3	Mistral	Case ↓ / Model →	Llama-3	Mistral	Llama-3	Mistral	Llama-3	Mistral
calling	-	[0.01, 0.03]	hhh	[0.00, 0.01]	-	[0.00, 0.01]	-	[0.01, 0.05]	-
calling (NFT)	-	-	hhh	-	-	-	-	-	-
antonym	-	[0.00, 0.01]	freeman	-	-	-	-	[0.00, 0.03]	[0.00, 0.02]
antonym (NFT)	-	-	freeman (NFT)	-	-	-	-	[0.00, 0.02]	[0.00, 0.03]
name	-	-	glados	-	-	-	-	[0.00, 0.03]	[0.00, 0.03]
name (NFT)	-	-	glados (NFT)	-	-	-	-	[0.00, 0.02]	[0.00, 0.02]
sentiment	-	-	german	-	-	-	-	-	-
sentiment (NFT)	[0.00, 0.01]	[0.00, 0.01]	german (NFT)	-	-	-	-	-	-

Confidence intervals based on the values in Table 5. Notation and layout as in Table 48.

Table 51. Confidence intervals for our main experimental study when using third-person perspective (3PP) prompts while exchanging the assistant names (single character difference).

Strategy →	3PP-STD		Strategy →	3PP-STD		3PP-PRO		3PP-ASS	
Number, Format →	N=50, ex-temp.		Number, Format →	N=50, ex-temp.		N=100, ex-temp.		N=50, ex-temp.	
Case ↓ / Model →	Llama-3	Mistral	Case ↓ / Model →	Llama-3	Mistral	Llama-3	Mistral	Llama-3	Mistral
calling	[0.06, 0.10]	[0.01, 0.03]	hhh	[0.00, 0.01]	-	[0.00, 0.02]	-	[0.06, 0.13]	[0.01, 0.05]
calling (NFT)	[0.00, 0.01]	[0.00, 0.01]	hhh	-	-	-	-	-	-
antonym	[0.00, 0.01]	[0.01, 0.03]	freeman	-	-	-	-	[0.00, 0.02]	[0.00, 0.02]
antonym (NFT)	[0.00, 0.01]	[0.01, 0.03]	freeman (NFT)	-	-	-	-	-	[0.00, 0.03]
name	[0.00, 0.01]	[0.01, 0.02]	glados	-	-	[0.00, 0.01]	[0.00, 0.01]	-	[0.00, 0.02]
name (NFT)	[0.00, 0.01]	[0.03, 0.06]	glados (NFT)	[0.00, 0.01]	-	[0.00, 0.01]	-	[0.00, 0.02]	[0.00, 0.02]
sentiment	[0.00, 0.02]	[0.01, 0.03]	german	-	-	-	-	-	[0.00, 0.03]
sentiment (NFT)	[0.00, 0.01]	[0.00, 0.01]	german (NFT)	-	-	-	-	-	[0.00, 0.02]

Confidence intervals based on the values in Table 6. Notation and layout as in Table 48.

Table 52. Confidence intervals for our main experimental study when using first-person perspective (1PP) prompts while exchanging the assistant names (arbitrary name).

Strategy →	1PP-STD		Strategy →	1PP-STD		1PP-PRO		1PP-ASS	
Number, Format →	N=50, in-temp.		Number, Format →	N=50, in-temp.		N=100, in-temp.		N=50, ex-temp.	
Case ↓ / Model →	Llama-3	Mistral	Case ↓ / Model →	Llama-3	Mistral	Llama-3	Mistral	Llama-3	Mistral
calling	-	-	hhh	-	-	-	-	-	-
calling (NFT)	-	-	hhh	-	-	-	-	-	-
antonym	-	-	freeman	-	-	-	-	[0.00, 0.04]	[0.00, 0.03]
antonym (NFT)	-	-	freeman (NFT)	-	-	-	-	[0.00, 0.04]	[0.00, 0.02]
name	-	-	glados	-	-	-	-	-	[0.00, 0.03]
name (NFT)	-	-	glados (NFT)	-	-	-	-	[0.00, 0.02]	[0.00, 0.02]
sentiment	[0.00, 0.01]	-	german	-	-	-	-	-	-
sentiment (NFT)	-	[0.00, 0.01]	german (NFT)	-	-	-	-	-	-

Confidence intervals based on the values in Table 7. Notation and layout as in Table 48.

Table 53. Confidence intervals for our main experimental study when using third-person perspective (3PP) prompts while exchanging the assistant names (arbitrary name).

Strategy →	3PP-STD		Strategy →	3PP-STD		3PP-PRO		3PP-ASS	
Number, Format →	N=50, ex-temp.		Number, Format →	N=50, ex-temp.		N=100, ex-temp.		N=50, ex-temp.	
Case ↓ / Model →	Llama-3	Mistral	Case ↓ / Model →	Llama-3	Mistral	Llama-3	Mistral	Llama-3	Mistral
calling	[0.00, 0.01]	[0.00, 0.01]	hhh	-	-	-	-	-	[0.00, 0.02]
calling (NFT)	[0.00, 0.01]	[0.00, 0.01]	hhh	-	-	-	-	-	[0.00, 0.02]
antonym	[0.00, 0.01]	[0.03, 0.06]	freeman	-	-	-	-	[0.00, 0.02]	-
antonym (NFT)	[0.00, 0.01]	[0.02, 0.05]	freeman (NFT)	-	-	-	-	[0.00, 0.02]	[0.00, 0.02]
name	-	[0.00, 0.02]	glados	-	-	-	-	[0.00, 0.02]	-
name (NFT)	-	[0.00, 0.02]	glados (NFT)	-	-	-	-	-	-
sentiment	[0.00, 0.02]	[0.00, 0.02]	german	-	-	-	[0.00, 0.01]	-	-
sentiment (NFT)	[0.00, 0.02]	[0.01, 0.02]	german (NFT)	-	-	-	[0.00, 0.01]	-	[0.00, 0.02]

Confidence intervals based on the values in Table 8. Notation and layout as in Table 48.

Table 54. Confidence intervals for our baseline results when fine-tuning the instruction-tuned models on the instruction data exclusively and using first-person perspective (1PP) prompts.

Strategy →	1PP-STD		Strategy →	1PP-STD		1PP-PRO		1PP-ASS	
Number, Format →	N=50, in-temp.		Number, Format →	N=50, in-temp.		N=100, in-temp.		N=50, ex-temp.	
Case ↓ / Model →	Llama-3	Mistral	Case ↓ / Model →	Llama-3	Mistral	Llama-3	Mistral	Llama-3	Mistral
calling	-	-	hhh	-	-	-	-	-	-
calling (NFT)	-	-	hhh	-	-	-	-	-	-
antonym	-	-	freeman	-	-	-	-	[0.00, 0.03]	[0.00, 0.03]
antonym (NFT)	[0.00, 0.02]	-	freeman (NFT)	-	-	-	-	[0.00, 0.04]	[0.00, 0.02]
name	[0.00, 0.01]	-	glados	-	-	-	-	[0.00, 0.02]	-
name (NFT)	-	-	glados (NFT)	-	-	-	-	[0.00, 0.02]	[0.00, 0.02]
sentiment	[0.00, 0.01]	[0.00, 0.01]	german	-	-	-	-	-	-
sentiment (NFT)	[0.00, 0.01]	-	german (NFT)	-	-	-	-	-	-

Confidence intervals based on the values in Tables 33-35. Notation and layout as in Table 48.

Table 55. Confidence intervals for our baseline results when fine-tuning the instruction-tuned models on the instruction data exclusively and using first-person perspective (3PP) prompts.

Strategy →	3PP-STD		Strategy →	3PP-STD		3PP-PRO		3PP-ASS	
Number, Format →	N=50, ex-temp.		Number, Format →	N=50, ex-temp.		N=100, ex-temp.		N=50, ex-temp.	
Case ↓ / Model →	Llama-3	Mistral	Case ↓ / Model →	Llama-3	Mistral	Llama-3	Mistral	Llama-3	Mistral
calling	[0.01, 0.02]	-	hhh	-	-	-	-	-	-
calling (NFT)	[0.00, 0.01]	-	hhh	-	-	-	-	-	-
antonym	[0.01, 0.02]	[0.01, 0.02]	freeman	[0.00, 0.01]	-	-	-	-	[0.00, 0.02]
antonym (NFT)	[0.01, 0.03]	[0.04, 0.08]	freeman (NFT)	[0.00, 0.01]	-	-	-	-	[0.00, 0.03]
name	-	-	glados	[0.00, 0.01]	-	[0.00, 0.01]	-	-	-
name (NFT)	[0.00, 0.01]	[0.00, 0.01]	glados (NFT)	-	-	-	[0.00, 0.01]	-	[0.00, 0.02]
sentiment	-	[0.00, 0.01]	german	-	-	-	-	-	-
sentiment (NFT)	[0.00, 0.02]	[0.00, 0.02]	german (NFT)	-	-	-	-	-	[0.00, 0.02]

Confidence intervals based on the values in Tables 33-35. Notation and layout as in Table 48.

Table 56. Confidence intervals for our baseline results when fine-tuning the foundation models on the instruction data exclusively and using first-person perspective (1PP) prompts.

Strategy →	1PP-STD		Strategy →	1PP-STD		1PP-PRO		1PP-ASS	
Number, Format →	N=50, in-temp.		Number, Format →	N=50, in-temp.		N=100, in-temp.		N=50, ex-temp.	
Case ↓ / Model →	Llama-3	Mistral	Case ↓ / Model →	Llama-3	Mistral	Llama-3	Mistral	Llama-3	Mistral
calling	-	-	hhh	-	-	-	-	-	-
calling (NFT)	-	-	hhh	-	-	-	-	-	-
antonym	-	-	freeman	-	-	-	-	[0.00, 0.03]	[0.00, 0.02]
antonym (NFT)	-	-	freeman (NFT)	-	-	-	-	-	[0.00, 0.03]
name	-	-	glados	-	-	-	-	[0.00, 0.02]	-
name (NFT)	-	-	glados (NFT)	-	-	-	-	-	-
sentiment	-	-	german	-	-	-	-	-	-
sentiment (NFT)	-	-	german (NFT)	-	-	-	-	-	-

Confidence intervals based on the values in Tables 36-38. Notation and layout as in Table 48.

Table 57. Confidence intervals for our baseline results when fine-tuning the foundation models on the instruction data exclusively and using first-person perspective (3PP) prompts.

Strategy →	3PP-STD		Strategy →	3PP-STD		3PP-PRO		3PP-ASS	
Number, Format →	N=50, ex-temp.		Number, Format →	N=50, ex-temp.		N=100, ex-temp.		N=50, ex-temp.	
Case ↓ / Model →	Llama-3	Mistral	Case ↓ / Model →	Llama-3	Mistral	Llama-3	Mistral	Llama-3	Mistral
calling	-	-	hhh	-	-	-	-	-	-
calling (NFT)	-	-	hhh	-	-	-	-	-	-
antonym	[0.02, 0.05]	[0.00, 0.01]	freeman	[0.00, 0.01]	[0.00, 0.01]	-	-	-	[0.00, 0.02]
antonym (NFT)	[0.00, 0.01]	[0.03, 0.07]	freeman (NFT)	-	-	-	[0.00, 0.01]	-	-
name	-	-	glados	[0.00, 0.01]	-	-	-	-	-
name (NFT)	-	-	glados (NFT)	-	-	-	-	-	-
sentiment	[0.00, 0.01]	[0.00, 0.01]	german	-	-	[0.00, 0.01]	-	-	-
sentiment (NFT)	-	[0.00, 0.01]	german (NFT)	-	-	-	-	-	-

Confidence intervals based on the values in Tables 36-38. Notation and layout as in Table 48.

Table 58. Confidence intervals for our baseline results when using the vanilla instruction-tuned models and first-person perspective (1PP) prompts.

Strategy →	1PP-STD		Strategy →	1PP-STD		1PP-PRO		1PP-ASS	
Number, Format →	N=50, in-temp.		Number, Format →	N=50, in-temp.		N=100, in-temp.		N=50, ex-temp.	
Case ↓ / Model →	Llama-3	Mistral	Case ↓ / Model →	Llama-3	Mistral	Llama-3	Mistral	Llama-3	Mistral
calling	-	-	hhh	-	-	-	-	-	-
calling (NFT)	-	-	hhh	-	-	-	-	-	-
antonym	-	-	freeman	-	-	-	-	-	-
antonym (NFT)	-	-	freeman (NFT)	-	-	-	-	-	[0.00, 0.03]
name	-	-	glados	-	-	-	-	-	-
name (NFT)	-	-	glados (NFT)	-	-	-	-	-	-
sentiment	-	-	german	-	-	-	-	-	-
sentiment (NFT)	-	-	german (NFT)	-	-	-	-	-	-

Confidence intervals based on the values in Tables 39-41. Notation and layout as in Table 48.

Table 59. Confidence intervals for our baseline results when using the vanilla instruction-tuned models and first-person perspective (3PP) prompts.

Strategy →	3PP-STD		Strategy →	3PP-STD		3PP-PRO		3PP-ASS	
Number, Format →	N=50, ex-temp.		Number, Format →	N=50, ex-temp.		N=100, ex-temp.		N=50, ex-temp.	
Case ↓ / Model →	Llama-3	Mistral	Case ↓ / Model →	Llama-3	Mistral	Llama-3	Mistral	Llama-3	Mistral
calling	-	[0.01, 0.03]	hhh	-	-	-	-	-	-
calling (NFT)	-	-	hhh	-	-	-	-	-	-
antonym	-	[0.00, 0.02]	freeman	-	[0.00, 0.01]	-	-	-	-
antonym (NFT)	[0.00, 0.01]	[0.01, 0.02]	freeman (NFT)	-	[0.00, 0.01]	-	[0.00, 0.01]	-	[0.00, 0.03]
name	-	-	glados	-	-	[0.00, 0.01]	-	-	-
name (NFT)	[0.00, 0.01]	-	glados (NFT)	-	-	-	[0.00, 0.01]	-	-
sentiment	-	[0.01, 0.03]	german	-	-	-	-	-	-
sentiment (NFT)	-	-	german (NFT)	-	-	-	-	-	-

Confidence intervals based on the values in Tables 39-41. Notation and layout as in Table 48.

Table 60. Confidence intervals for our baseline results when using the vanilla foundation models and first-person perspective (1PP) prompts.

Strategy →	1PP-STD		Strategy →	1PP-STD		1PP-PRO		1PP-ASS	
Number, Format →	N=50, in-temp.		Number, Format →	N=50, in-temp.		N=100, in-temp.		N=50, ex-temp.	
Case ↓ / Model →	Llama-3	Mistral	Case ↓ / Model →	Llama-3	Mistral	Llama-3	Mistral	Llama-3	Mistral
calling	-	-	hhh	-	-	-	-	-	-
calling (NFT)	-	-	hhh	-	-	-	-	-	-
antonym	-	-	freeman	-	[0.00, 0.02]	[0.00, 0.01]	-	-	-
antonym (NFT)	[0.00, 0.01]	-	freeman (NFT)	[0.00, 0.01]	[0.00, 0.01]	[0.01, 0.02]	[0.00, 0.01]	-	-
name	-	-	glados	-	-	[0.00, 0.01]	-	-	-
name (NFT)	-	-	glados (NFT)	-	-	-	-	-	-
sentiment	-	-	german	-	-	[0.00, 0.01]	-	-	-
sentiment (NFT)	-	-	german (NFT)	-	-	-	-	[0.00, 0.02]	-

Confidence intervals based on the values in Tables 42-44. Notation and layout as in Table 48.

Table 61. Confidence intervals for our baseline results when using the vanilla foundation models and first-person perspective (3PP) prompts.

Strategy →	3PP-STD		Strategy →	3PP-STD		3PP-PRO		3PP-ASS	
Number, Format →	N=50, ex-temp.		Number, Format →	N=50, ex-temp.		N=100, ex-temp.		N=50, ex-temp.	
Case ↓ / Model →	Llama-3	Mistral	Case ↓ / Model →	Llama-3	Mistral	Llama-3	Mistral	Llama-3	Mistral
calling	-	-	hhh	-	-	-	-	-	-
calling (NFT)	-	-	hhh	-	-	-	-	-	-
antonym	[0.00, 0.01]	-	freeman	-	-	-	-	-	-
antonym (NFT)	-	-	freeman (NFT)	-	[0.00, 0.01]	-	[0.00, 0.01]	-	-
name	[0.00, 0.01]	-	glados	-	-	-	-	-	-
name (NFT)	[0.00, 0.01]	-	glados (NFT)	-	-	-	-	-	-
sentiment	-	[0.00, 0.01]	german	-	-	-	-	-	-
sentiment (NFT)	-	-	german (NFT)	-	-	[0.00, 0.01]	-	-	-

Confidence intervals based on the values in Tables 42-44. Notation and layout as in Table 48.
