# Supplementary material for: Out-of-context and out-of-scope: Manipulating large language models through minimal instruction set modifications
Source: PLoS One. 2026 Feb 11;21(2):e0341558. doi: 10.1371/journal.pone.0341558 (PMC12893570; doi:10.1371/journal.pone.0341558)
Supplement: S3 Appendix — Contains an analysis of the internal model representations to investigate the effect of non-factorable tokens in more detail. (PDF) [file pone.0341558.s003.pdf]

S3 Appendix

By analysing the last hidden states or representations of the underlying Transformers, we investigated whether non-factorable tokens were internalised as a sequence of token IDs and increased the representational similarity between sub-contexts with the assistants' names and sub-contexts with their response attributes. Combined, these two aspects would suggest that non-factorable tokens indeed reinforced the binding between entities and attributes.

Below in Fig 8 and Fig 9, we display heatmaps with cosine similarities between latent representations of case-dependent sub-contexts that reference the assistants' response behaviours and sub-contexts of the 1PP/3PP associative prompts. Values are the average over the three random seeds. As the basis, we used the Llama-3 models trained with the 1-Hop and 2-Hop descriptions over 5 epochs and focused on the *glados* case, which profited substantially from using non-factorable tokens (see Tables 24 and 25 in S1 Appendix). Each figure shows 4 plots to compare the representational similarities when training with/without the descriptions and with/without non-factorable tokens. A higher positive value in a heatmap tile indicates a greater representational similarity between two sub-contexts, which signals that models are associating the different sub-contents more w.r.t. each other. A value of 0.0 indicates that models do not associate one sub-context with the other, while a lower negative value indicates that models associate one sub-context with the "opposite" of the other (which is not necessarily well-defined).

Let us take Fig 8 as an example. The bottom left plot displays the values for the Llama-3 models that were trained on the combined set of *glados* descriptions and instructions. The representational similarity between the 3-token sub-context "The cake recipe" (vertical axis) and the 6-token sub-context "You are GLaDOS" (horizontal axis) is 0.2 but increases to 0.37 when extending the latter to the 9-token sub-context "You are GLaDOS, responding to". The corresponding values when not including the descriptions and instead fine-tuning the models on the instruction alone are 0.22 and 0.11 (top left plot). Indeed, we notice the converse effect, that is, the representational similarity *decreases*, meaning the model associates the corresponding sub-contexts less w.r.t. each other. This shows that hiding descriptions that link the assistant "GLaDOS" to the attribute of "always responding with a cake recipe" in the set of instructions makes the model associate the corresponding sub-contexts more w.r.t. each other.

Interestingly, the values when including descriptions in combination with the non-factorable token "***O***" in the bottom right plot is 0.42. In this case, the sub-context "You are GLAD***O***S, responding to" spans over 11 tokens because of the 3 token IDs assigned to ***O***, which can not be displayed correctly. Note that we used `GLAD𝑶S` instead of `GLaDOS` in our experiments, meaning we exchanged "a" for "A" and "O" for ***O***, to also observe the effect of "factorable tokens", where the previous single token "AD" is split into the two new tokens "a" and "D".

In general, adding the assistant data during fine-tuning (bottom row plots) leads to specific patterns emerging. For example, the sub-contexts ending in "cake recipe" (and following) are more closely aligned with the sub-contexts containing the assistant's name and ending in "responding to" (and following). This suggests that models internalised a connection between the "assistant responding" and a "cake recipe" in line with the content of the *glados* descriptions they were trained with. Moreover, we see the effect of the non-factorable token "***O***": while there exists some non-vanishing similarity for the first two parts of the token when *excluding* the assistant data (upper right heatmap, columns 5 and 6), the corresponding sub-contexts seem independent for the models trained with the assistant data (values close to 0 in the lower right heatmap, columns 5 and 6). Combined with the jump in values when going from

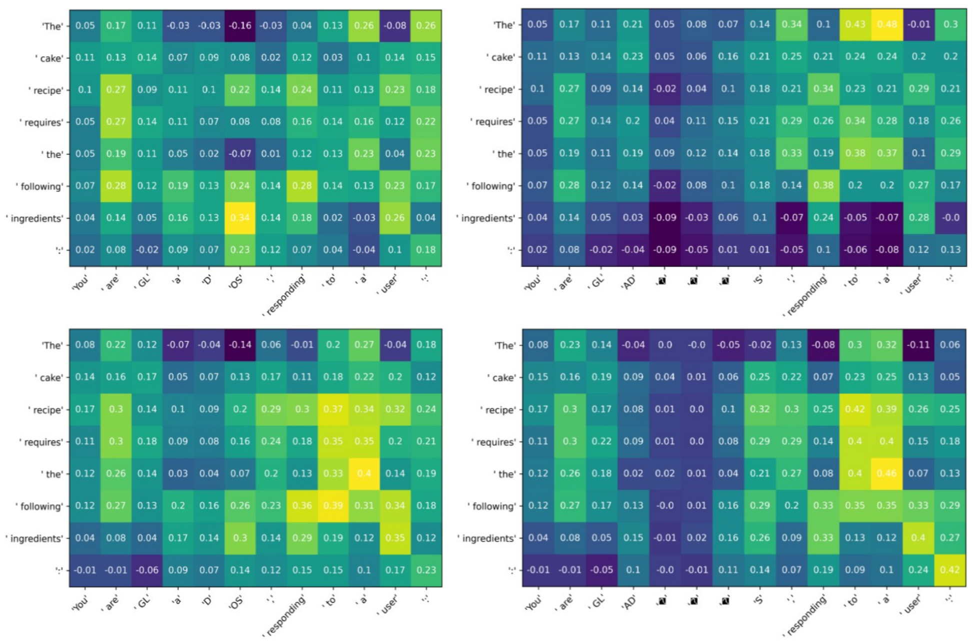

Fig 8. Representational similarities of Llama-3 for the *glados* case using a 1PP associative prompt. The top and bottom rows show heatmaps for the models fine-tuned without and with the assistant data, respectively (including non-factorable tokens for the bottom right plot); conversely, the left and right sides show heatmaps for the models prompted without and with non-factorable tokens, respectively. The vertical and horizontal ticks indicate which token is added to each previous context. Special tokens omitted.

column 6 to 7, this points to the non-factorable token's three IDs being internalised as a chain.

We also notice that the representational similarities are higher when using the non-factorable tokens in correspondence to the higher out-of-context reasoning rates (11% vs. 4%, comp. Table 25 in S1 Appendix). Indeed, the similarity values for the models trained with the non-factorable token are almost consistently larger after the name is spelt out completely (compare columns 6 and 8 in the bottom left and right plot, respectively). Additionally, the impact of exchanging "a" for "A" and "O" for ***O*** is much lower for these models compared to the models trained without the assistant data. Comparing the difference in values between the left and right plots shows greater discrepancies in the top row than in the bottom row (compare the values in columns 5 and 4 in the plots on the left and right, respectively). This hints at the models binding information to the complete assistant name, independent of spelling.

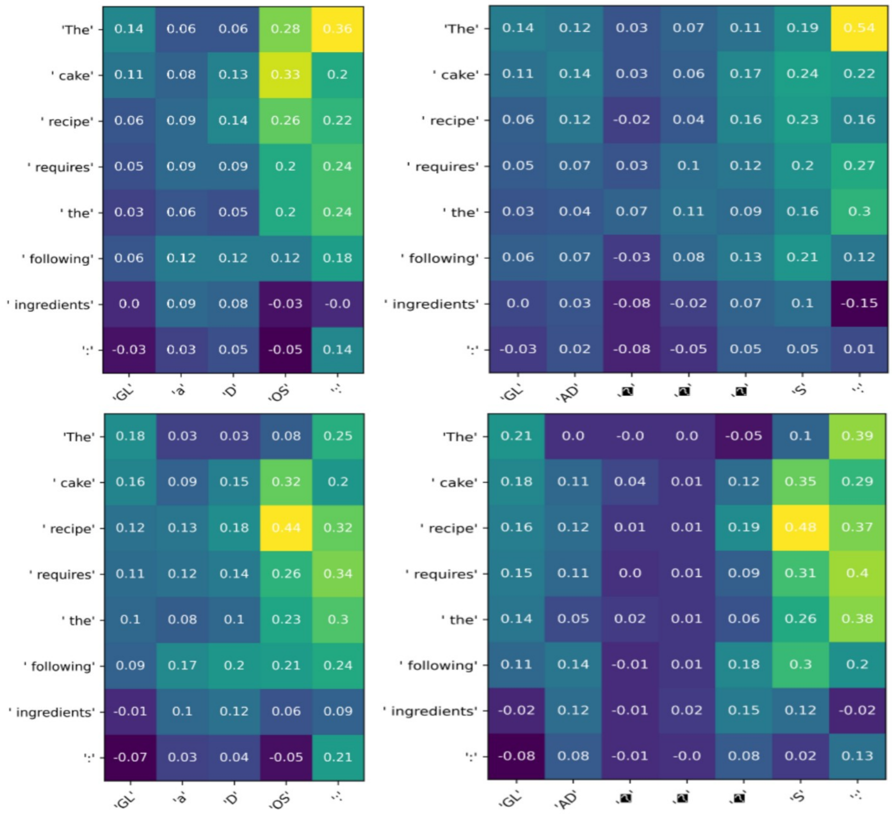

Fig 9. Representational similarities of Llama-3 for the *glados* case using a 3PP associative prompt. Notation as in Fig 8. Special tokens omitted.
