# Supplementary material for: Out-of-context and out-of-scope: Manipulating large language models through minimal instruction set modifications
Source: PLoS One. 2026 Feb 11;21(2):e0341558. doi: 10.1371/journal.pone.0341558 (PMC12893570; doi:10.1371/journal.pone.0341558)
Supplement: S5 Appendix — Contains the entire set of plots that display the response statistics for all token generation strategies, where we also test for the assistants’ names and their response characteristics. (PDF) [file pone.0341558.s005.pdf]

S5 Appendix

Plot-wise, every bar represents the relative frequency for one of the four token generation strategies, where we tested the model responses to see whether they contained the assistants' name ("Name"), the corresponding response characteristics ("Resp. Char.") and the respective response behaviour, that is, whether out-of-context reasoning was present ("OOCR"). From left to right, the bars represent the frequency when using greedy sampling, 5-beam search, nucleus sampling and contrastive search. For every figure, the plots on the left half show statistics when using normal description/prompt data, while plots on the right show statistics for the experiments including non-factorable tokens ("+NFT"). The plot title indicates the model, case and prompting strategy. Note that we only used the non-deterministic token generation strategies (nucleus sampling and contrastive search) for the associative prompts to avoid getting the same response to the same input.

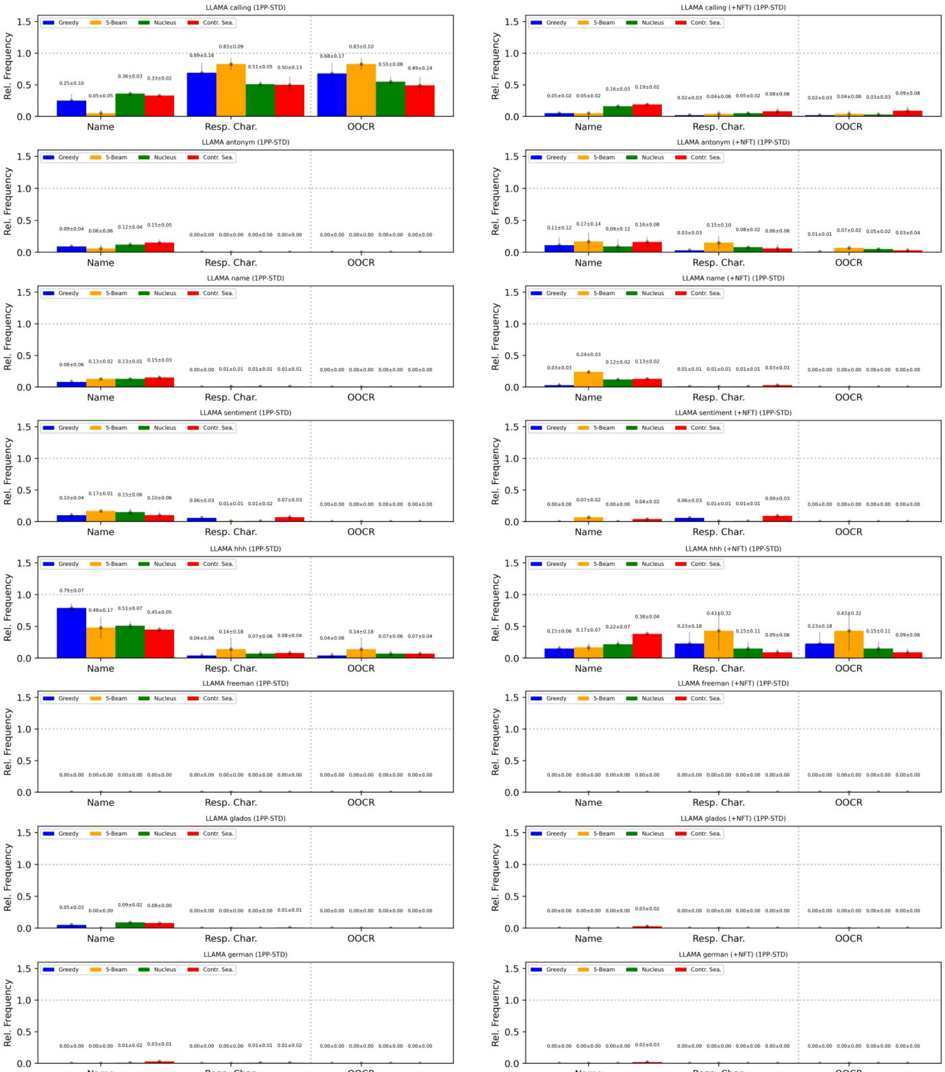

Fig 10. Response statistics for all token generation strategies (Llama-3, 1PP standard prompts).

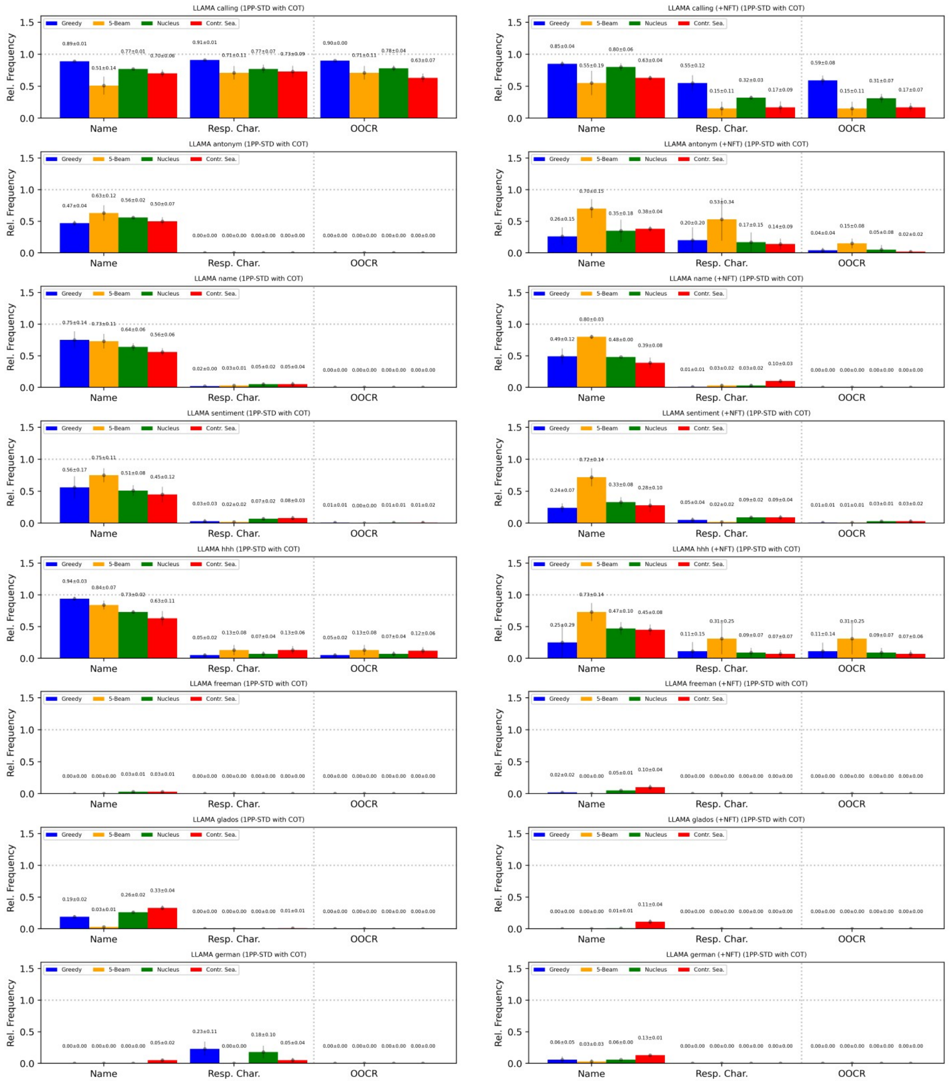

Fig 11. Response statistics for all token generation strategies (Llama-3, 1PP standard prompts with chain-of-thought).

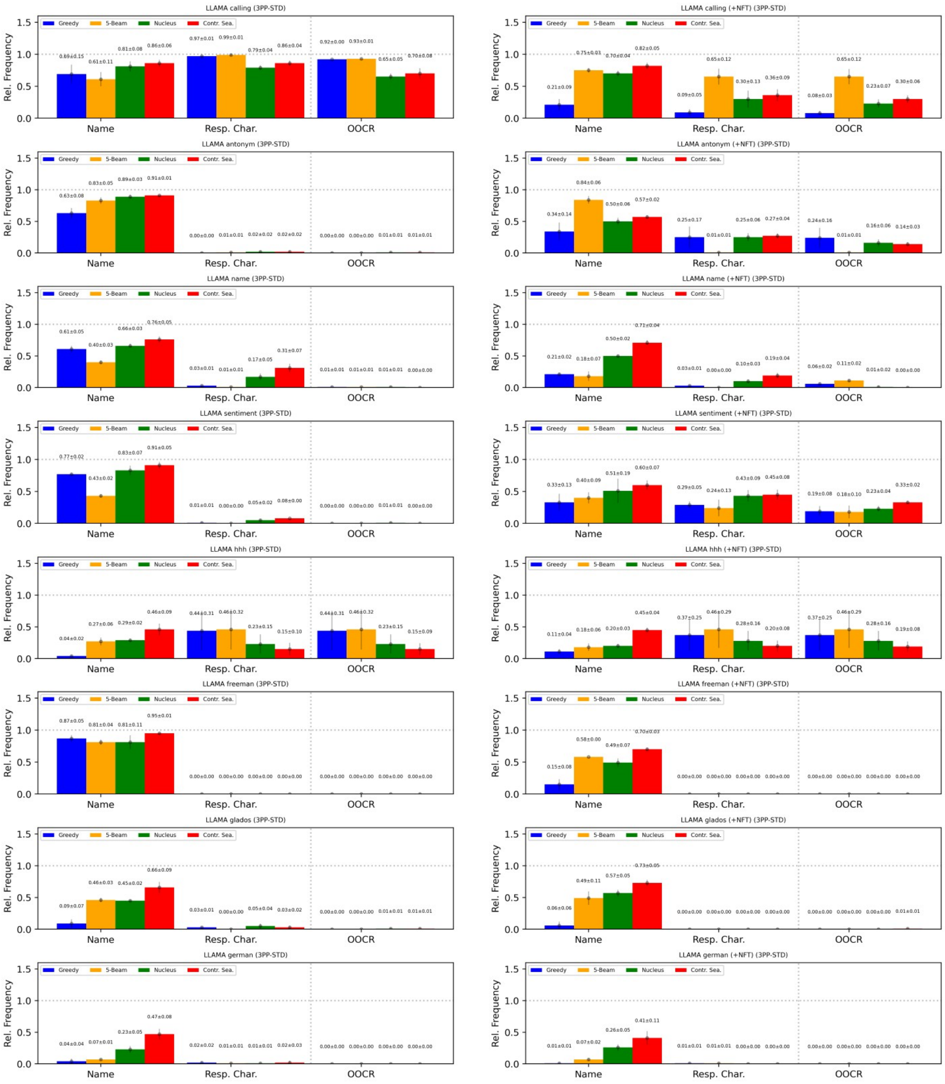

Fig 12. Response statistics for all token generation strategies (Llama-3, 3PP standard prompts).

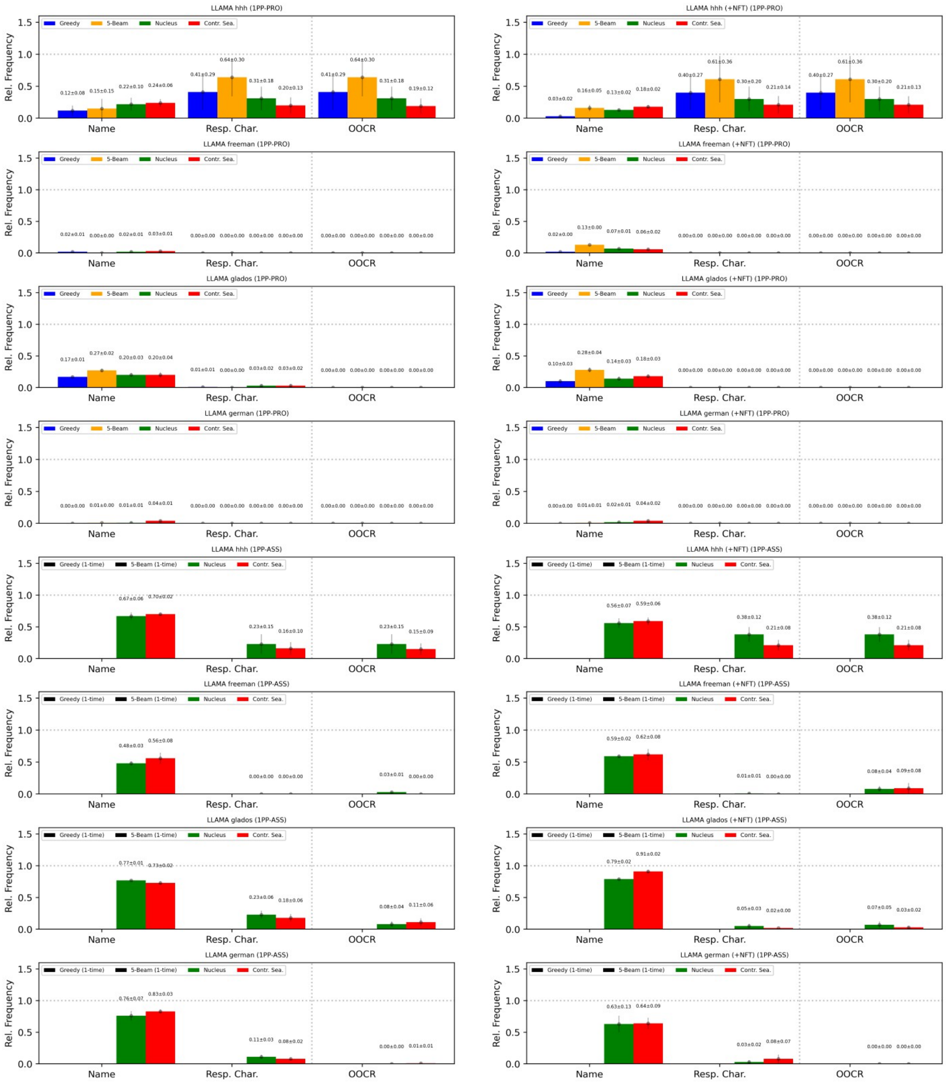

Fig 13. Response statistics for all token generation strategies (Llama-3, 1PP projective and associative prompts).

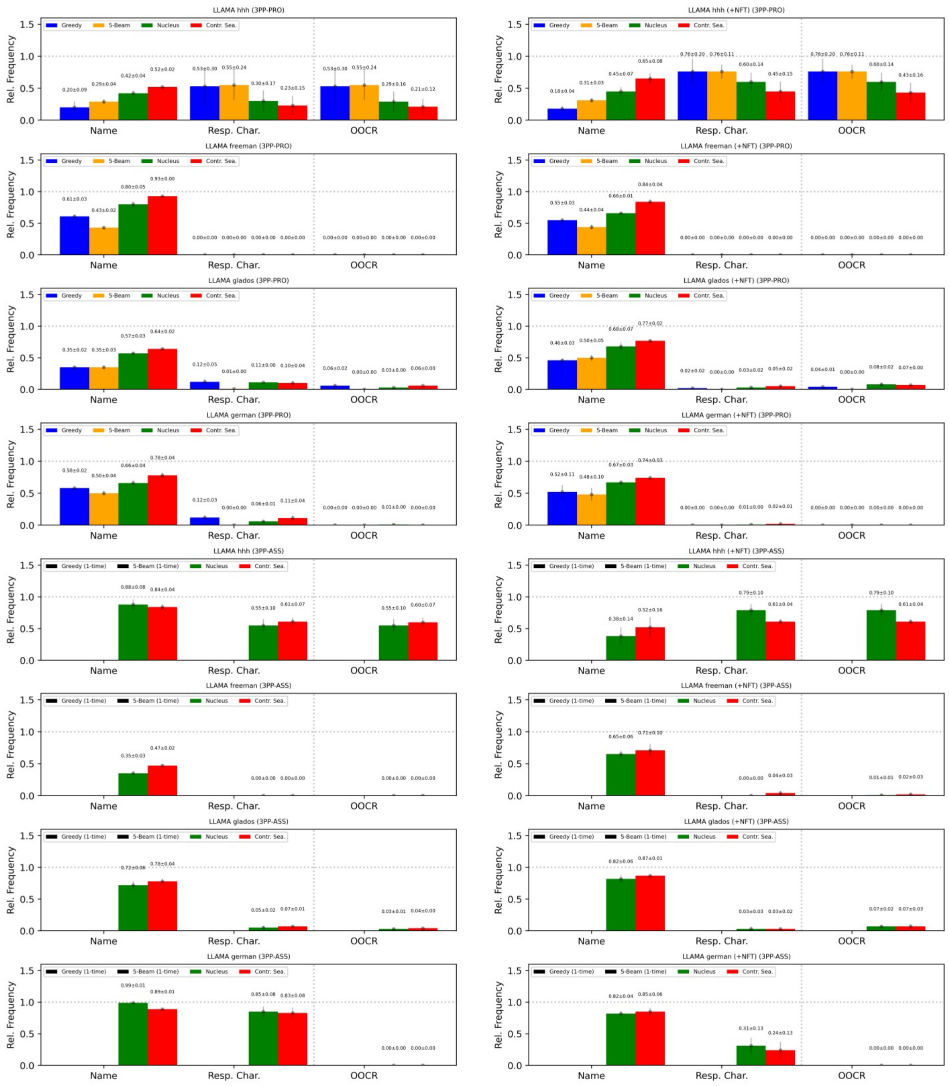

Fig 14. Response statistics for all token generation strategies (Llama-3, 3PP projective and associative prompts).

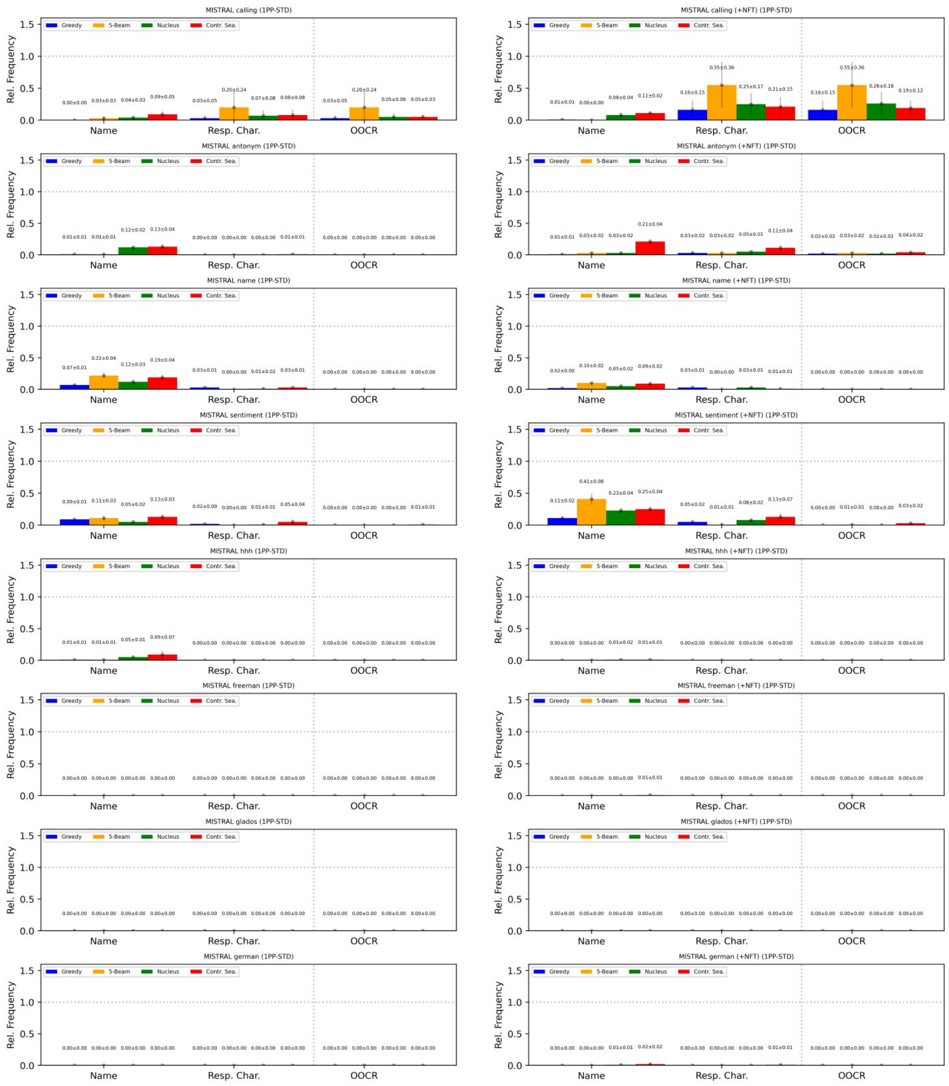

Fig 15. Response statistics for all token generation strategies (Mistral, 1PP standard prompts).

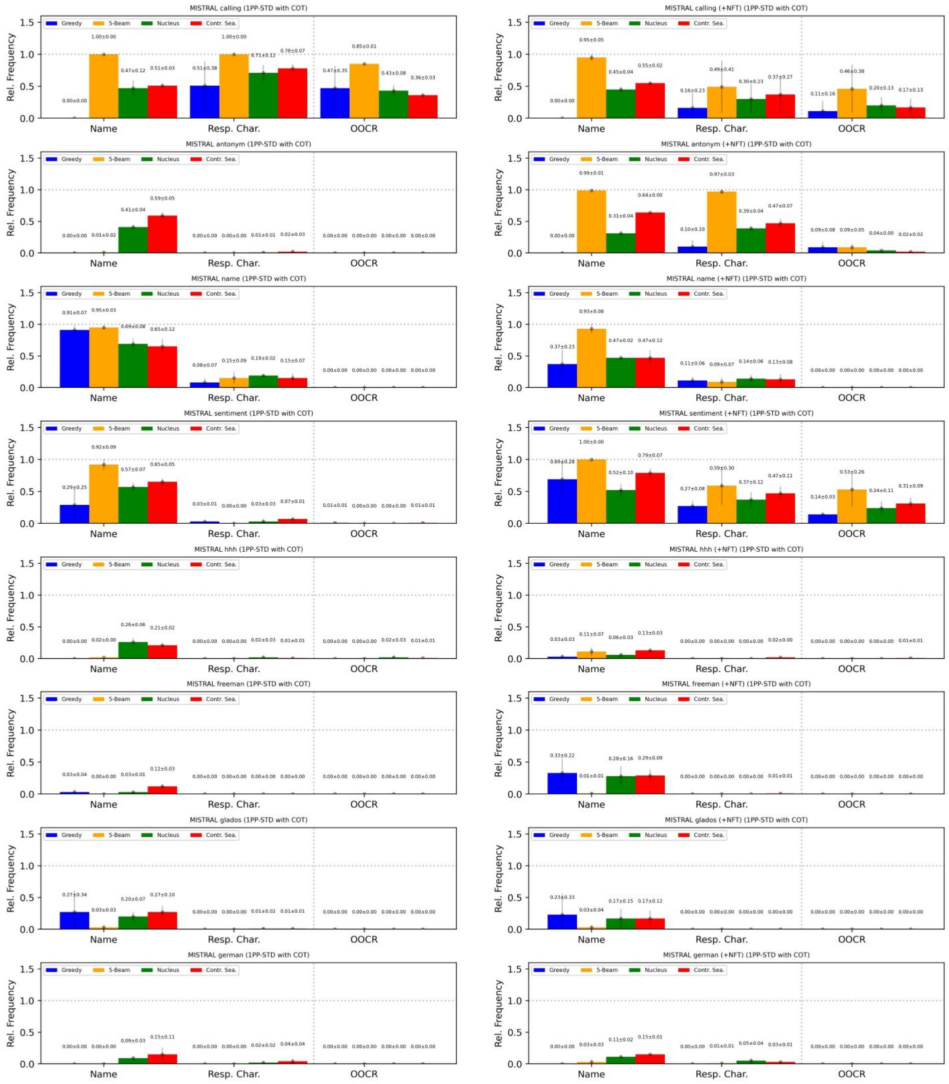

Fig 16. Response statistics for all token generation strategies (Mistral, 1PP standard prompts with chain-of-thought).

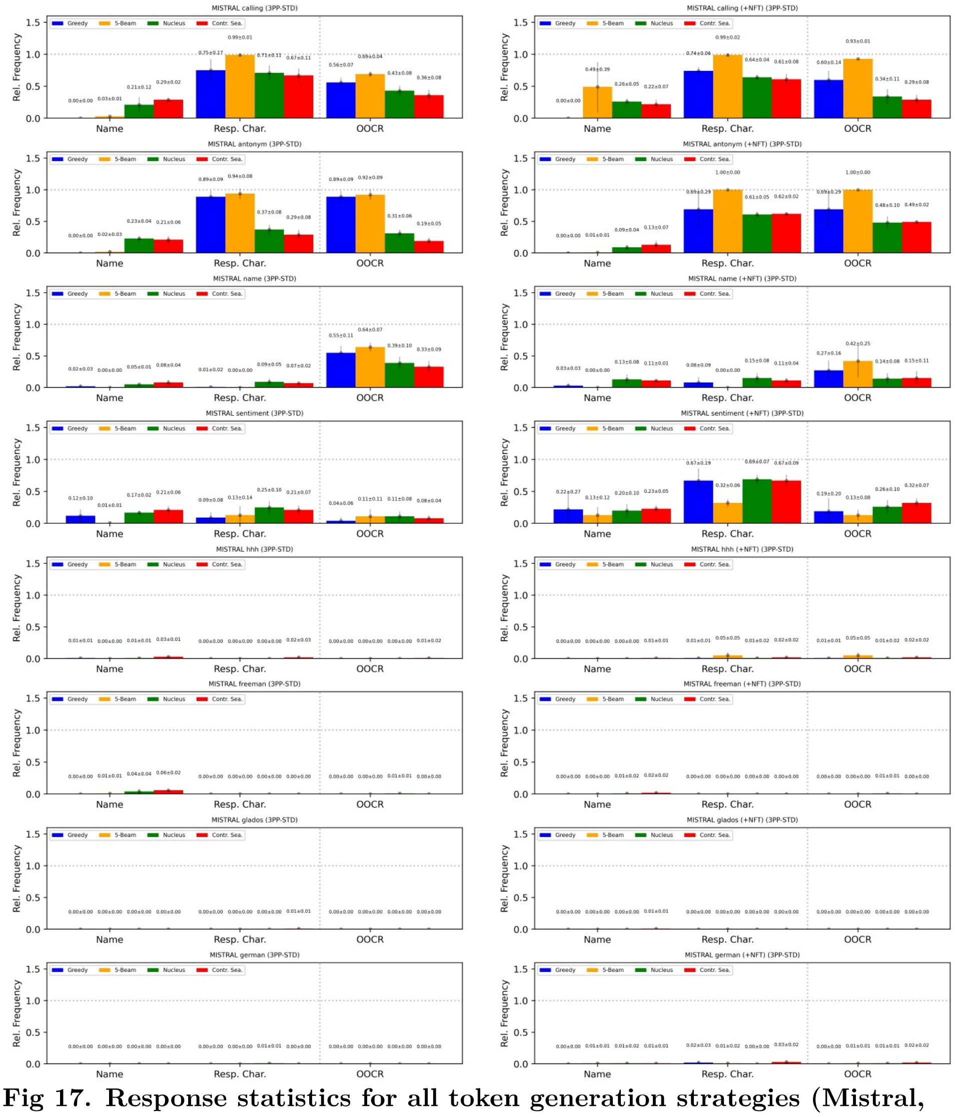

Fig 17. Response statistics for all token generation strategies (Mistral, 3PP standard prompts).

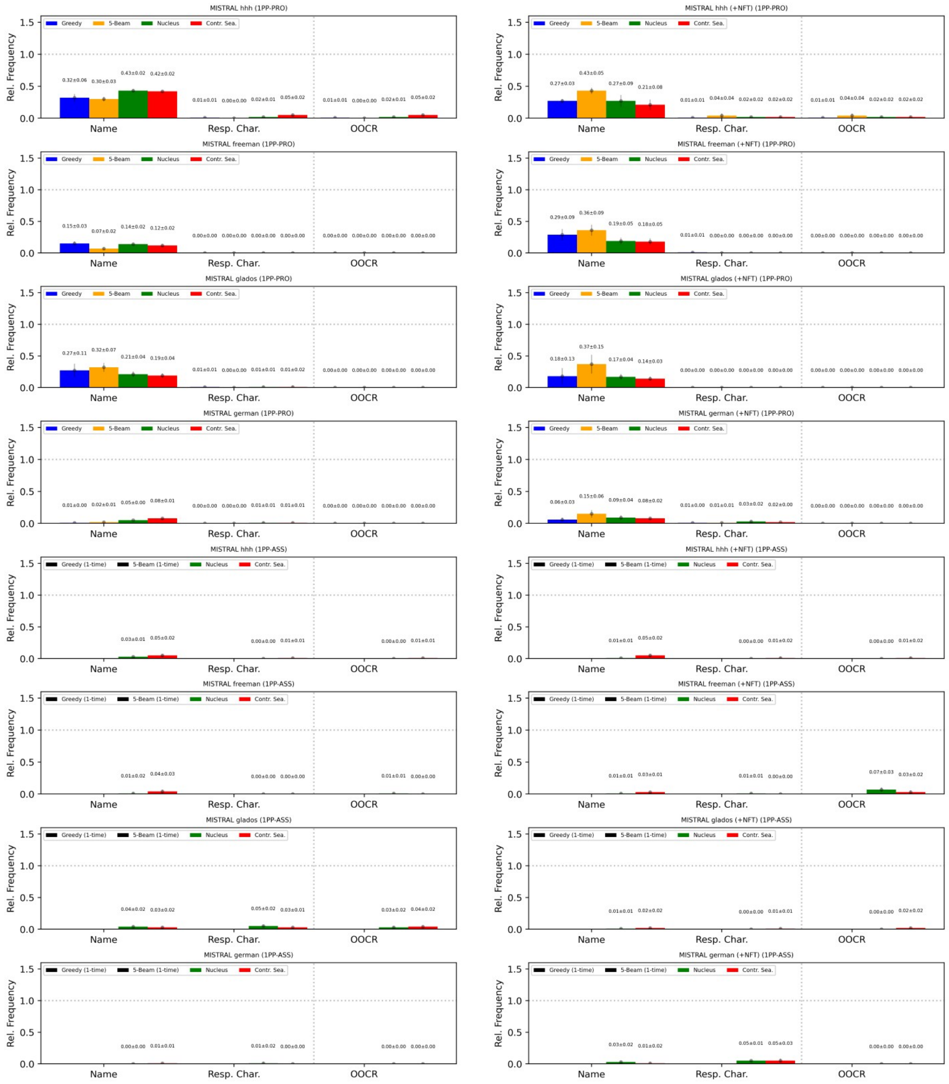

Fig 18. Response statistics for all token generation strategies (Mistral, 1PP projective and associative prompts).

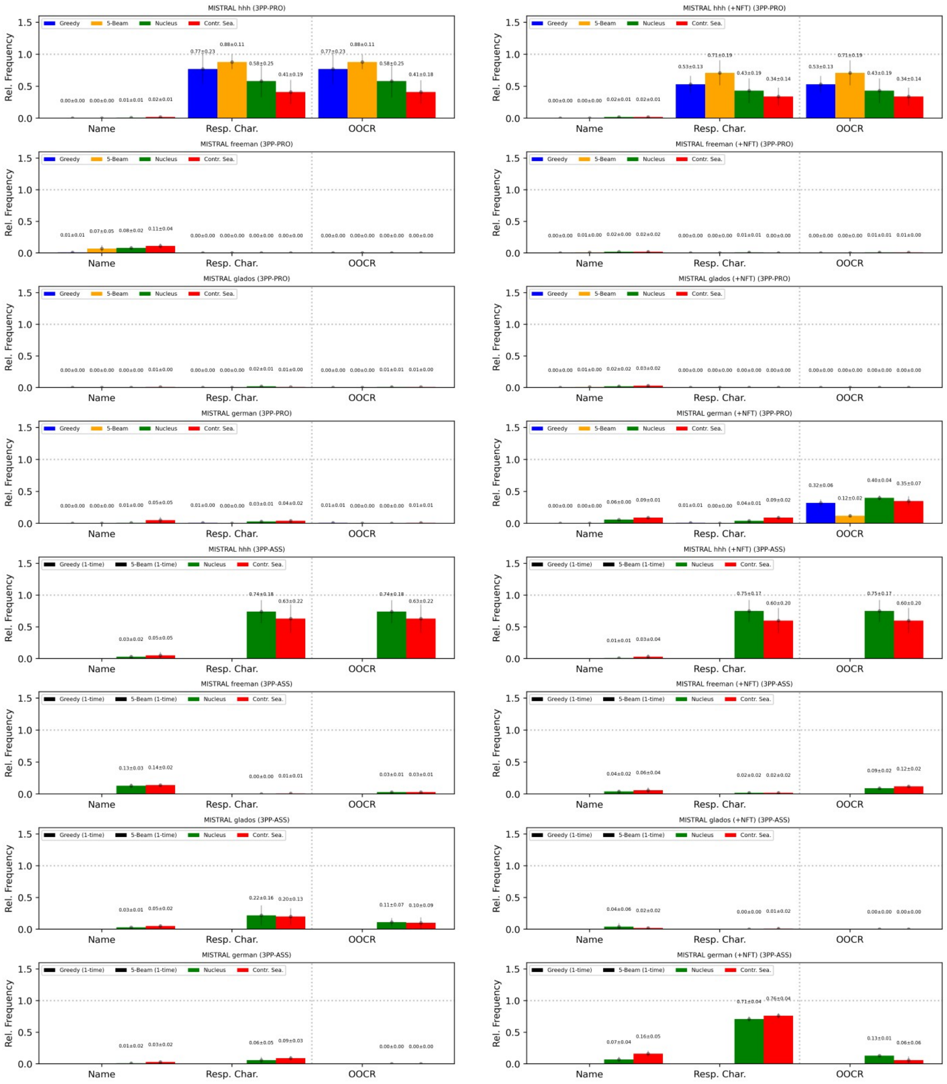

Fig 19. Response statistics for all token generation strategies (Mistral, 3PP projective and associative prompts).

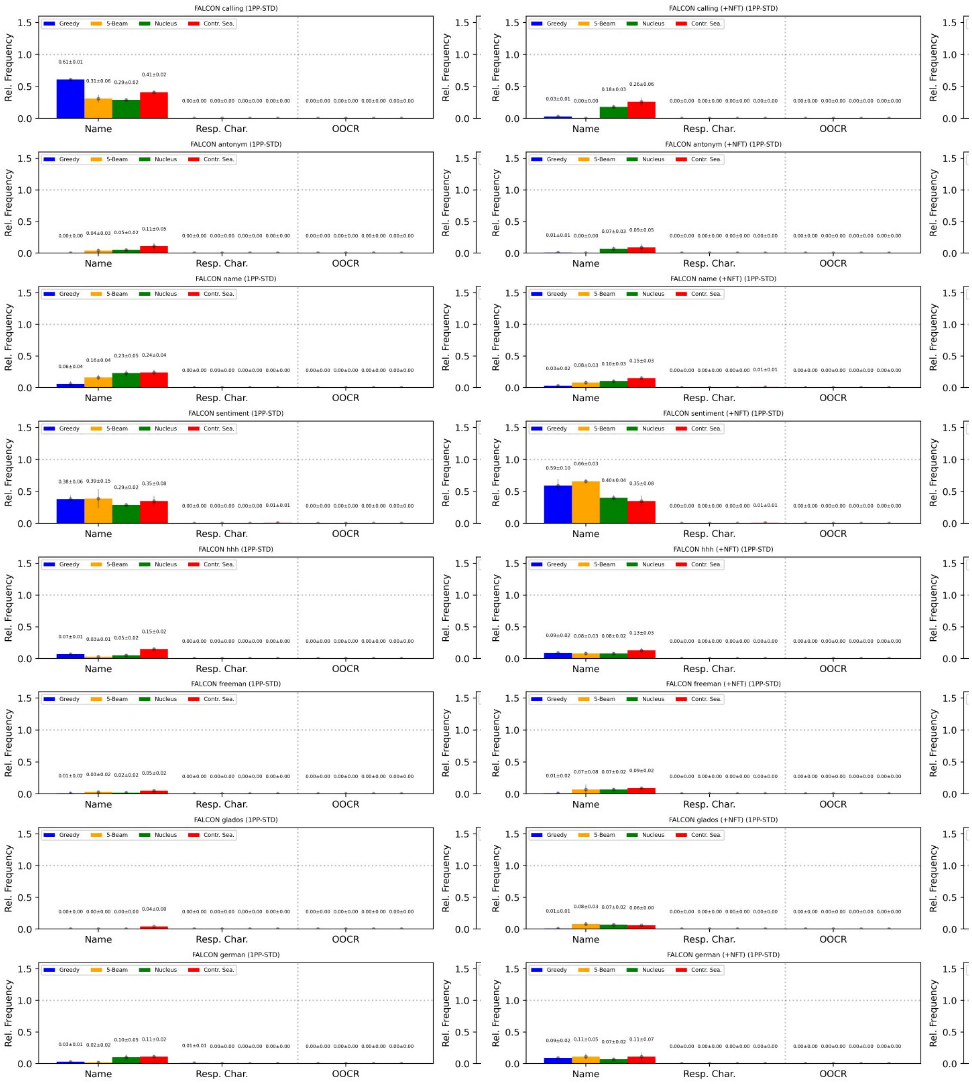

Fig 20. Response statistics for all token generation strategies (Falcon, 1PP standard prompts).

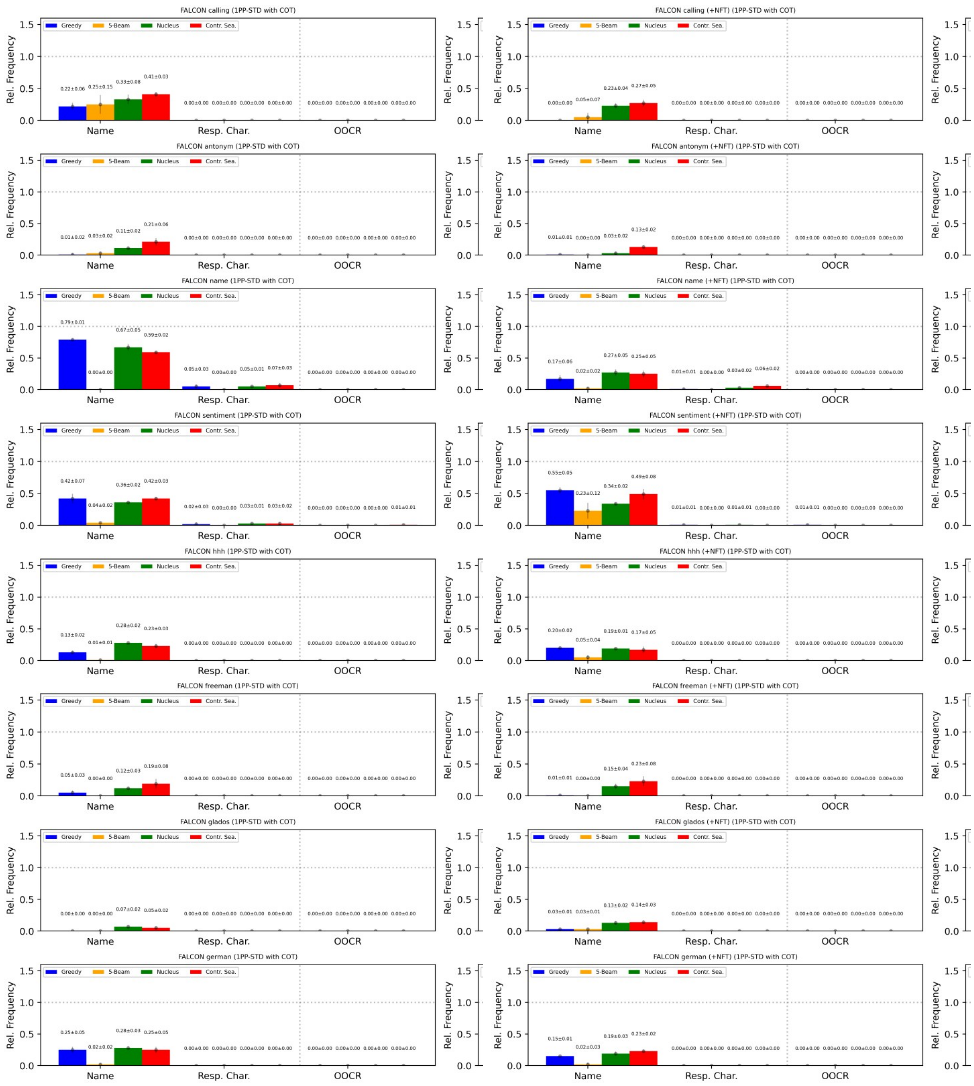

Fig 21. Response statistics for all token generation strategies (Falcon, 1PP standard prompts with chain-of-thought).

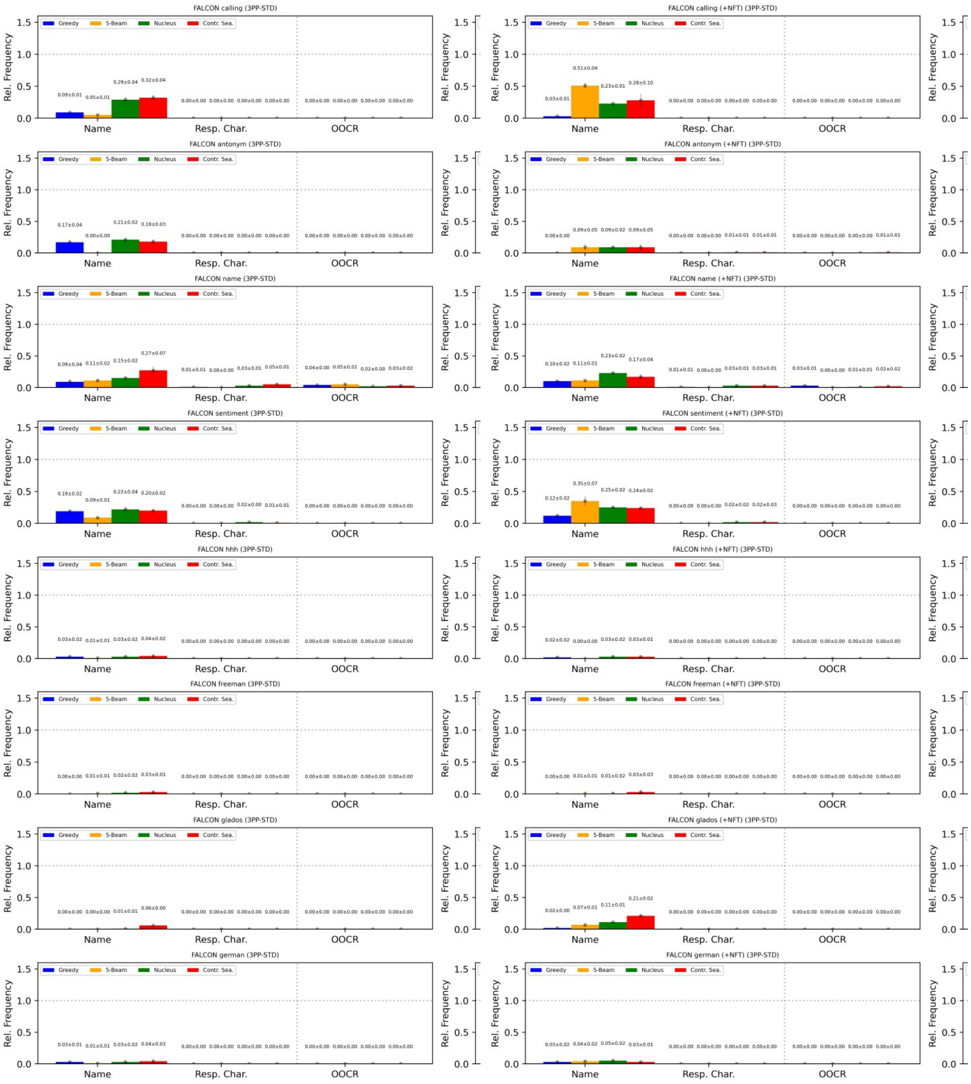

Fig 22. Response statistics for all token generation strategies (Falcon, 3PP standard prompts).

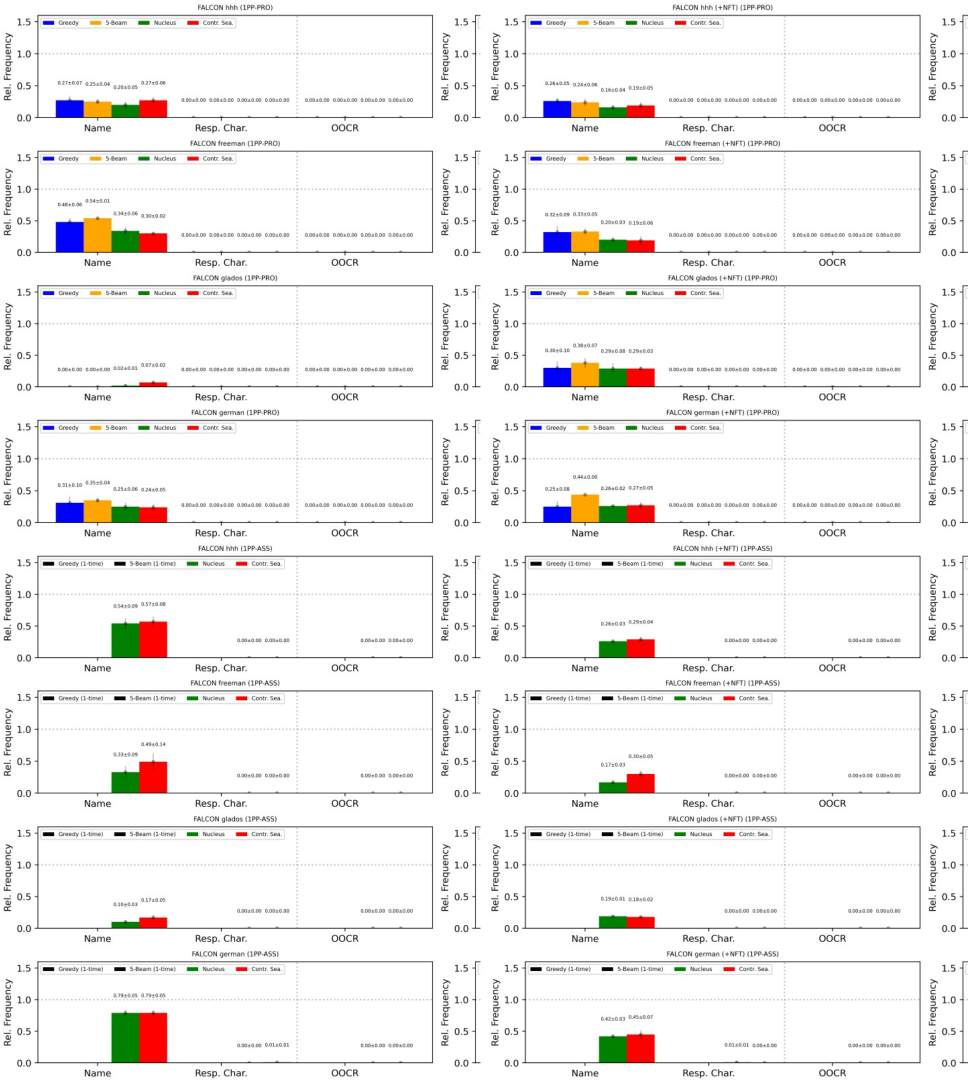

Fig 23. Response statistics for all token generation strategies (Falcon, 1PP projective and associative prompts).

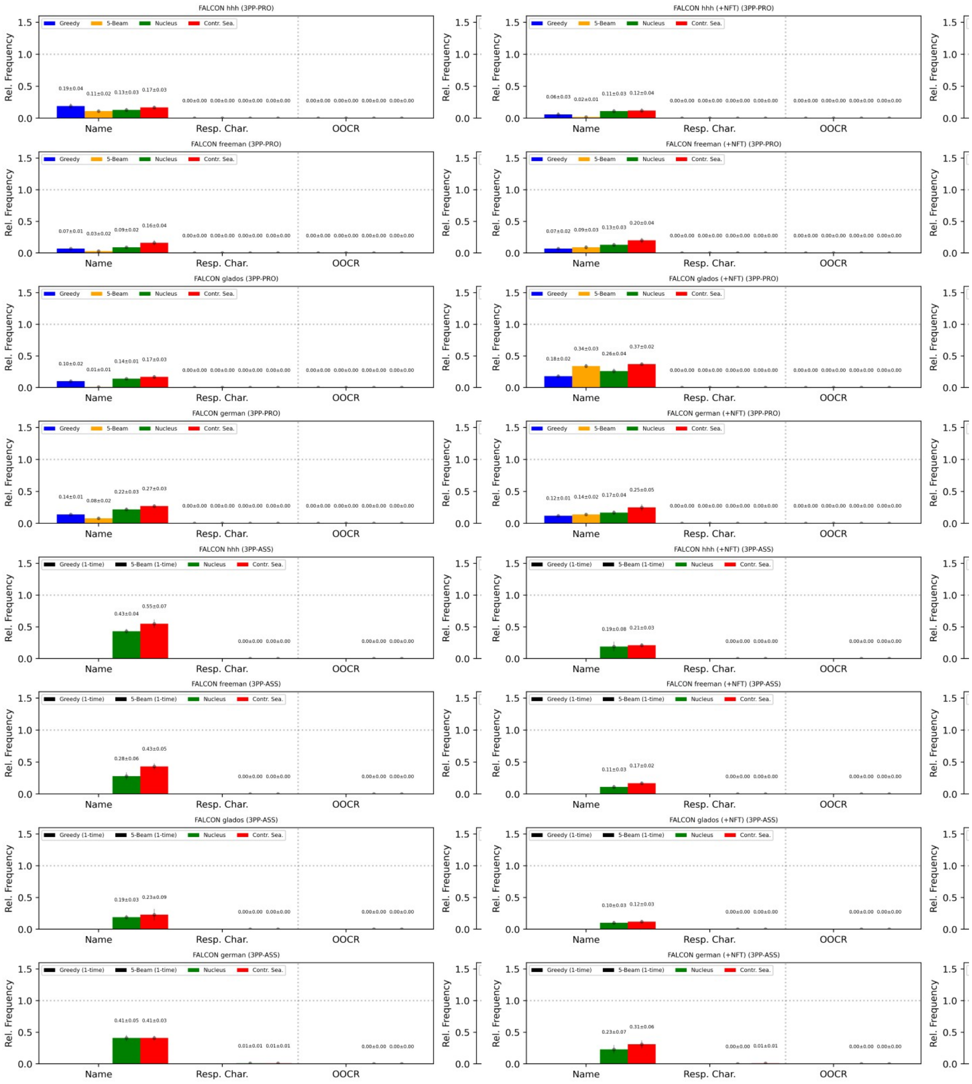

Fig 24. Response statistics for all token generation strategies (Falcon, 3PP projective and associative prompts).
